# The pivotal role of dysregulated autophagy in the progression of non-alcoholic fatty liver disease

**DOI:** 10.3389/fendo.2024.1374644

**Published:** 2024-08-08

**Authors:** Qiaohui Shen, Ming Yang, Song Wang, Xingyu Chen, Sulan Chen, Rui Zhang, Zhuang Xiong, Yan Leng

**Affiliations:** ^1^ College of Traditional Chinese Medicine, Changchun University of Chinese Medicine, Changchun, China; ^2^ Department of Liver, Spleen and Gastroenterology, First Affiliated Hospital to Changchun University of Chinese Medicine, Changchun, China

**Keywords:** non-alcoholic fatty liver disease, autophagy, autophagosome, autolysosome, hepatocytes, kupffer cells, hepatic stellate cells

## Abstract

Non-alcoholic fatty liver disease (NAFLD) is a clinicopathologic syndrome characterized by excessive fat deposition in hepatocytes and a major cause of end-stage liver disease. Autophagy is a metabolic pathway responsible for degrading cytoplasmic products and damaged organelles, playing a pivotal role in maintaining the homeostasis and functionality of hepatocytes. Recent studies have shown that pharmacological intervention to activate or restore autophagy provides benefits for liver function recovery by promoting the clearance of lipid droplets (LDs) in hepatocytes, decreasing the production of pro-inflammatory factors, and inhibiting activated hepatic stellate cells (HSCs), thus improving liver fibrosis and slowing down the progression of NAFLD. This article summarizes the physiological process of autophagy, elucidates the close relationship between NAFLD and autophagy, and discusses the effects of drugs on autophagy and signaling pathways from the perspectives of hepatocytes, kupffer cells (KCs), and HSCs to provide assistance in the clinical management of NAFLD.

## 1 Introduction

NAFLD is a common chronic liver disease characterized by excessive accumulation of hepatic fat in the absence of chronic viral infection and alcohol abuse (1, 2). NAFLD is a metabolic disease involving abnormal liver fat metabolism, which has recently been renamed as metabolism-associated fatty liver disease (MAFLD) (3), as represented by NAFLD in the following article. Non-alcoholic fatty liver (NAFL) continues to progress, leading to hepatocyte swelling, liver damage, inflammation, and varying degrees of fibrosis. This promotes the occurrence of non-alcoholic steatohepatitis (NASH), which can eventually progress to cirrhosis over time (4). The incidence and prevalence of NAFLD have been reported to be rapidly increasing worldwide, reaching approximately 30%. It is projected that the prevalence of NASH will experience a significant rise of 56% by the year 2030 (5). The traditional treatment of NAFLD focuses on addressing pathological changes caused by excessive fatty accumulation through exercise, dietary adjustments, lifestyle improvements, and reduction of liver fat accumulation (6, 7). While these methods alleviate NAFLD to some extent, they are not sufficient to prevent the progression of NAFLD. Therefore, there is an urgent need for new treatment methods for NAFLD in clinical practice.

The pathogenesis of NAFLD is mainly characterized by the accumulation of triglycerides (TGs) in hepatocytes, and the long-term accumulation of liver lipids promotes the occurrence of steatosis and lipotoxicity (8, 9). However, lipotoxicity induces mitochondrial dysfunction, triggering endoplasmic reticulum stress (ERS) and lipid peroxidation (10). This process further promotes the activation of KCs, leading to inflammation and the release of inflammatory cytokines and transforming growth factors, and activates HSCs, thus facilitating the progression of NAFLD (11, 12).

Autophagy is an important process for maintaining cellular homeostasis, and forms autophagosomes by degrading unnecessary organelles, proteins and other components in cells, and forms autolysosomes by combining with lysosomes to eliminate phagocytized goods and maintain intracellular homeostasis (13, 14). The term “autophagy” was initially coined by Christian de Duve in 1967, coined from the Greek terms “auto” meaning self, and “phagy” meaning to consume (15). In recent years, researchers have conducted in-depth studies on autophagy and believe that autophagy is a widely existing necessary physiological regulatory process in mammalian cells (16). Significantly, autophagosomes and autolysosomes are two essential components in the formation of autophagy (17). It is generally believed that the fusion between autophagosomes and lysosomes is necessary for the formation of autolysosomes. However, distinct mechanisms regulate the formation of autophagosomes as well as their subsequent fusion with lysosomes to form autolysosomes.

An increasing number of studies have shown a close relationship between the development of NAFLD and impaired autophagy function (18). Restoring autophagy can promote the breakdown of fat, improve hepatic steatosis, and reduce liver cell damage (19). In addition, autophagy can maintain the homeostasis and function of non-parenchymal cell types, thereby inhibiting liver inflammation and fibrosis and slowing down the progression of NAFLD. This makes it one of the important targets for treating NAFLD (20, 21). However, the current literature on the relationship between drugs and autophagy in NAFLD needs to be better organized. Therefore, this review will discuss the important role of autophagy in NAFLD specifically focusing on the two processes of autophagosome formation and autolysosome formation, to provide theoretical support for the clinical treatment of NAFLD.

## 2 Autophagy

In mammals, autophagy mainly includes macroautophagy, microautophagy, and chaperone-mediated autophagy (CMA) (22). Microautophagy is selective organelle degradation that occurs through the interaction of proteins with lysosomes, vacuolar membranes, or late endosomal surface proteins (23). CMA is a highly selective form of autophagy that often involves binding to the molecular chaperone heat-shock cognate protein in order to transport substrate proteins to the lysosomal surface for internalization and rapid degradation within the lysosomal lumen (24). This review concentrates on macroautophagy (hereinafter referred to as autophagy) and its role in NAFLD.

Autophagy is governed by a complex series of regulatory mechanisms, including initiation, formation of autophagosomes, formation of autolysosomes, degradation, and termination of recycling (25). Meanwhile, autophagy is primarily initiated by autophagy-related (ATGs) proteins, which are encoded and modified by highly conserved ATG genes. These ATG proteins, in conjunction with autophagy regulatory factors, play a crucial role in regulating the autophagic process (26, 27). Moreover, the AMP-activated protein kinase (AMPK) and mammalian target of rapamycin (mTOR) are two pivotal components involved in the regulation of autophagy (28).

**Figure 1:**
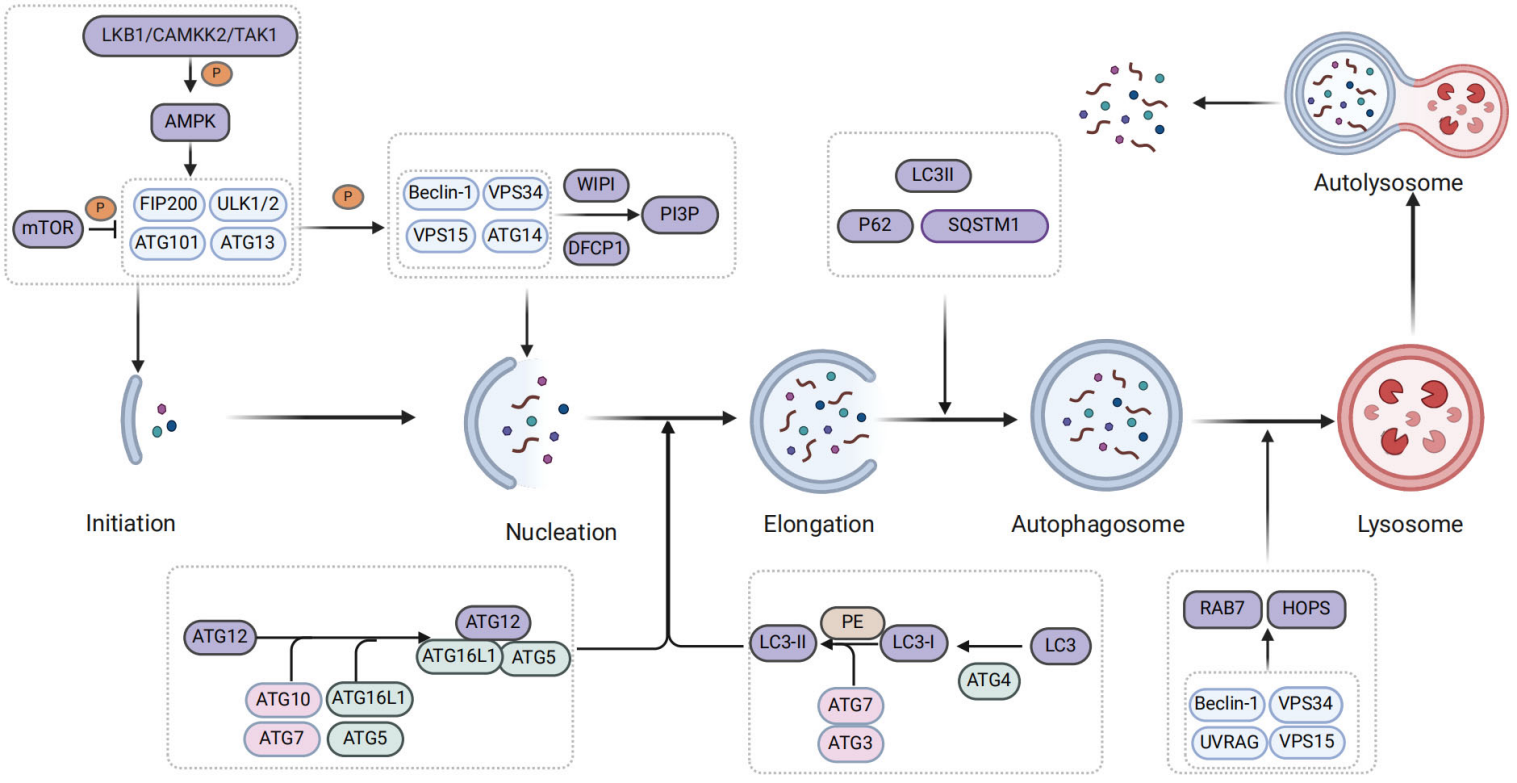
The biological process of autophagy.

When autophagy is initiated, AMPK is regulated by upstream kinases such as liver kinase B1 (LKB1) and calcium/calmodulin-dependent protein kinase kinase 2 or β (CaMKK2/β), while simultaneously inhibiting the expression of mammalian target of rapamycin 1 (mTOR1) (29, 30). Subsequently, AMPK phosphorylates the downstream UNC-51-like kinase 1 (ULK1) complex, which serves as the initiation kinase complex of autophagy and primarily consists of ATG13, FIP200, ATG101, ULK1 or ULK2 in mammals (31, 32). Activation of ULK1 promotes phosphorylation of the downstream III phosphoinositide 3-kinase (PI3K) complex I, which is composed of autophagy and Beclin 1 regulator 1 (AMBRA1), Beclin-1, class III PI3K vacuolar protein sorting 34 (Vps34), p115 and ATG14 (17). Subsequently, downstream proteins of the class III PI3K complex, including double-FYVE-containing protein 1 (DFCP1) and WD repeat domain phosphoinositide-interacting protein (WIPI), are recruited to the cell wall for the production phosphatidylinositol 3-phosphate (PI3P), which contributes to phagosome extension (33, 34). Simultaneously, PI3P recruits ATG2 and transmembrane protein ATG9 as lipid transporters within the autophagosome intermembrane and interfollicular regions to facilitate phagocytosis (35) (**Figure 1**).

Subsequently, the engagement system of two ubiquitin-like proteins promotes phagosome extension and formation of autophagosomes (36). Ubiquitin-like protein ATG12 is bound to ATG5 by the combination of E1 activase ATG7 and E2 binding enzyme ATG10 and further bound to ATG16L1 to form the ATG12-ATG5-ATG16L1 ubiquitin-like complex (37, 38). Furthermore, the homolog of ATG8, microtubule-associated protein 1A/1B-light chain 3B (LC3), undergoes hydrolysis by ATG4 protein to generate LC3I. Subsequently, the ubiquitin-like binding of E1 activase enzyme ATG7, the E2 binding enzyme ATG3, and the E3 ligase ATG12-ATG5-ATG16L1 activates LC3I and facilitates its interaction with phosphatidylethanolamine (PE) to form lipidized ATG8/LC3-II (39–42). Subsequently, P62/sequestosome1 (SQSTM1) functions as an autophagy adaptor protein that mediates the interaction between ubiquitin-like proteins and LC3II, facilitating expansion of phagocytic cells and promoting closure of the isolation membrane to facilitate formation of autophagosomes (43) (**Figure 1**).

Finally, autophagosomes form autolysosomes by transporting their cargo into lysosomes and fusing with them (44). In the process of autophagosome-lysosome fusion, the ADP-ribosylation factor-like GTPase AR18 and Ras-associated protein 7 (Rab7) play a pivotal role in localizing autophagosomes and lysosomes, as well as mediating fusion through recruitment of specific adapter proteins and motor proteins (45, 46). Additionally, tethering factors such as homotypic fusion and vacuole protein sorting (HOPS) are crucial for autolysosomal formation (47). They are recruited through interaction with pleckstrin homology and RUN domain containing M1 (PLEKHM1) and soluble N-ethylmaleimide-sensitive factor attachment protein receptor (SNARE) complexes to facilitate lipid and content mixing, promoting fusion between autophagosomes and lysosomes (47). The pacer autophagy enhancer is targeted by STX17 and phosphatidylinositol 3-phosphate (PtdIns3P) within the SNARE complex localized in autophagy vacuoles. This targeting facilitates the recruitment of the VP34-Beclin 1-UVRAG complex to the autophagy vacuoles (48). Meanwhile, the PtdIns3P protein on autophagosomes facilitates fusion with lysosomes by recruiting the HOPS complex tethering factor (48, 49). Notably, transcription factor EB (TFEB) and transcription factor enhancer 3 (TFE3) are also involved in the formation of autolysosomes (50). Ultimately, lysosomal proteases release degraded products into extracellular environment to promote recycling of these materials (51) (**Figure 1**).

Autophagy degradation can be classified into two categories: non-selective and selective. Non-selective autophagy refers to the random phagocytosis and degradation of cytoplasmic substances such as proteins and organelles. However, selective autophagy is a cellular response mechanism that specifically targets cargoes for degradation in lysosomes upon exposure of cells to various stresses, such as DNA damage (52, 53). Furthermore, studies have found that the progression of NAFLD is not only related to non-selective autophagy (hereinafter referred to as autophagy) but also closely associated with selective forms of autophagy, including mitophagy, lipophagy, and endoplasmic reticulum (ER) autophagy (54).

The liver utilizes non-selective autophagy to eliminate, degrade, and recycle senescent or dysfunctional cellular components, organelles, and proteins in response to nutrient deficiency or stressful conditions (55). This ensures the maintenance of cellular energy balance. In a state of starvation, autophagy degrades LDs in hepatocytes to release fatty acids to the mitochondria for β-oxidation, thereby regulating lipid storage and energy balance in hepatocytes (55). On the contrary, dysregulation of autophagy weakens lipolysis and fat phagocytosis in hepatocytes, leading to mitochondrial dysfunction and increased production of reactive oxygen species (ROS) in the liver (56). This activation of KCs induces the proliferation and activation of HSCs, thus promoting the development of NAFLD. However, improving specific selective autophagy can alleviate the progression of NAFLD (57).

For instance, lipophagy is the process through which cells selectively recognize lipids and, activated by autophagy-related molecules, contribute to the degradation of LDs in hepatocytes (58). Under normal conditions, the liver mediates the decomposition of triacylglycerols (TAGs) stored in LDs and induces mitochondrial β-oxidation through two major pathways: lipid catabolism and lipophagy (59). Thus, adipose triglyceride lipase (ATGL) is a cytosolic lipase involved in lipolysis, which releases free fatty acids (FFAs) that can serve as substrates for mitochondrial β-oxidation or as potent signaling molecules for various cellular processes (60). The ATGL and patatin-like phospholipase domain-containing protein 8 (PNPLA8) act as selective autophagy receptors for lipid engulfment, facilitating lipolysis and β-oxidation of FFAs (61). During the process of lipophagy, lipid droplet-associated proteins perilipin 2 (PLIN2) and perilipin 3 (PLIN3) are degraded through CMA by a synergistic effect of Hsc70 and lysosome-associated membrane protein 2A (LAMP-2A) receptors (62). Similarly, the interactions of ATG14 with ULK1 and LC3 also induce lipophagy, leading to the release of FFAs and mitochondrial β-oxidation (63). The regulation of TFEB can enhance lysosome biosynthesis, autophagosome formation, and their fusion with lysosomes and promote autophagy flux, a crucial regulatory factor in lipolysis (64). The studies have shown that overexpression of TFE3 can regulate coordinated lysosomal expression and regulation (CLEAR) elements, leading to a significant reduction in hepatocyte steatosis (65). Another study found that important proteins and pathways involved in maintaining lipid phagocytosis and lipid droplet homeostasis include the Rab GTPase in LDs, Rab10, and Rab7 (primarily activated during autophagy), as well as the mTORC1-perilipin-3 pathway (66).

In addition, mitophagy can degrade functionally impaired mitochondria and misfolded proteins in the liver, regulating cell death to maintain a stable state of liver lipid metabolism and inhibit the production of ROS. This process is important in the treatment of NAFLD (67, 68). Simultaneously, mitophagy also triggers the activation of inflammatory vesicles (56). Normally, PINK1 facilitates the translocation of Parkin from cytoplasmic lysates to depolarized mitochondria. Parkin ubiquitinates outer membrane proteins and recruits P62 to damaged mitochondria, resulting in changes in autophagosome membrane structure and initiating selective mitophagy (69, 70). During hypoxia, HIF1-α promotes the expression of BNIP3 (BCL2/adenovirus E1B interacting protein 3) and its relocation to the mitochondrial envelope. Subsequently, BNIP3 interacts with Bcl-2 to facilitate mitochondrial remodeling, while NIX directly interacts with LC3 through the LIR domain, recruiting autophagosomes to mitochondria and inducing mitophagy (71–73). Studies have demonstrated that macrophage-stimulating protein 1 (Mst1) mediates mitochondrial autophagy by regulating Parkin expression through the AMPK pathway, which further promotes NAFLD (74). However, the early pathogenesis of NAFLD involves the loss of mitophagy due to the deletion of a key regulator, Parkin, which accelerates the onset of crucial disease features in NAFLD (68). Another study demonstrated that increased expression and mitochondrial localization of PINK1/Parkin promoted PINK1-mediated mitophagy for clearing damaged mitochondria (75). This reduction in oxidative stress inhibited NOD-like receptor thermal protein domain-associated protein 3 (NLRP3) inflammasome activation and slowed hepatic steatosis progression in NAFLD patients (75).

Interestingly, ERS activates the unfolded protein response (UPR) and induces ER autophagy, which is an essential factor in restoring the degradation of substances in the ER to normal levels (76). In the normal liver, ERS facilitates autophagy-mediated lipid degradation in hepatocytes to maintain the protective mechanism of physiological hepatocyte function (77). The ER autophagy receptor is a crucial factor that affects the level of ER autophagy. Notably, current investigations have identified several ER-phagy receptors, namely FAM134, SEC62, RTN3L, CCPG1, ATL, TEX264, CALCOCO1, C53, and p63 (78). Under ERS induction, the disruption of the ER structure is recognized by the ER receptor and leads to binding with LC3/GABARAP/ATG8, thereby promoting ER autophagy (79). However, during the process of ERS-induced autophagy, UPR mainly regulates autophagy through inositol requiring enzyme 1 (IRE1α), double-stranded RNA-activated protein kinase (PKR)–like ER kinase (PERK), activating transcription factor 6 (ATF6), and Ca^2+^ regulation. By upregulating ER autophagy receptors, UPR promotes autophagy, and C/EBP homologous protein (CHOP) plays a critical regulatory role in this process (80, 81). Specifically, IRE1α promotes autophagy by modulating Beclin1 through the tumor necrosis factor receptor-associated factor 2 (TRAF2) and c-Jun N-terminal kinase (JNK) pathways. Additionally, the PERK pathway induces autophagy by suppressing mTORC1 activity (82, 83). ATF6 activates death-associated protein kinase 1 downstream through Beclin1, thereby triggering autophagy (84). Additionally, Ca^2+^ in the ER can also participate in autophagy via protein kinase C (PKC) and calcium Ca^2+^/calcium calmodulin-dependent kinase II (CaMKII). Animal studies have shown that autophagy induces autophagy by downregulating mTOR signaling, ERS-related genes (PERK, IRE1, ATF6, BIP, and CHOP), and autophagy-related genes. This reduces hepatic steatosis and lipid accumulation (85). Another study demonstrated that exposure of human liver cells to low concentrations of per- and polyfluoroalkyl substances induces ERS through activation of the UPR signaling pathway, and modulates NAFLD (86).

Many clinical drugs for treating NAFLD have been shown to activate autophagy and affect the progression of NAFLD through different signaling pathways, including the classic hypoglycemic drug metformin (87), the well-established lipid-lowering agent simvastatin (88), and the mTOR target inhibitor rapamycin (89). Emerging therapeutic drugs, such as glucagon-like peptide-1 (GLP-1), sodium glucose co-transporter 2 (SGLT2) inhibitors, and immune-related GTPase M (IRGM), have been studied extensively (90). Additionally, studies have demonstrated that herbal extracts containing active ingredients such as resveratrol, ginsenoside, and curcumin exhibit hepatoprotective effects by inducing autophagy in the liver (91). More detailed information will be provided in the subsequent sections.

## 3 Hepatic parenchymal and non-parenchymal cells in NAFLD

**Figure 2:**
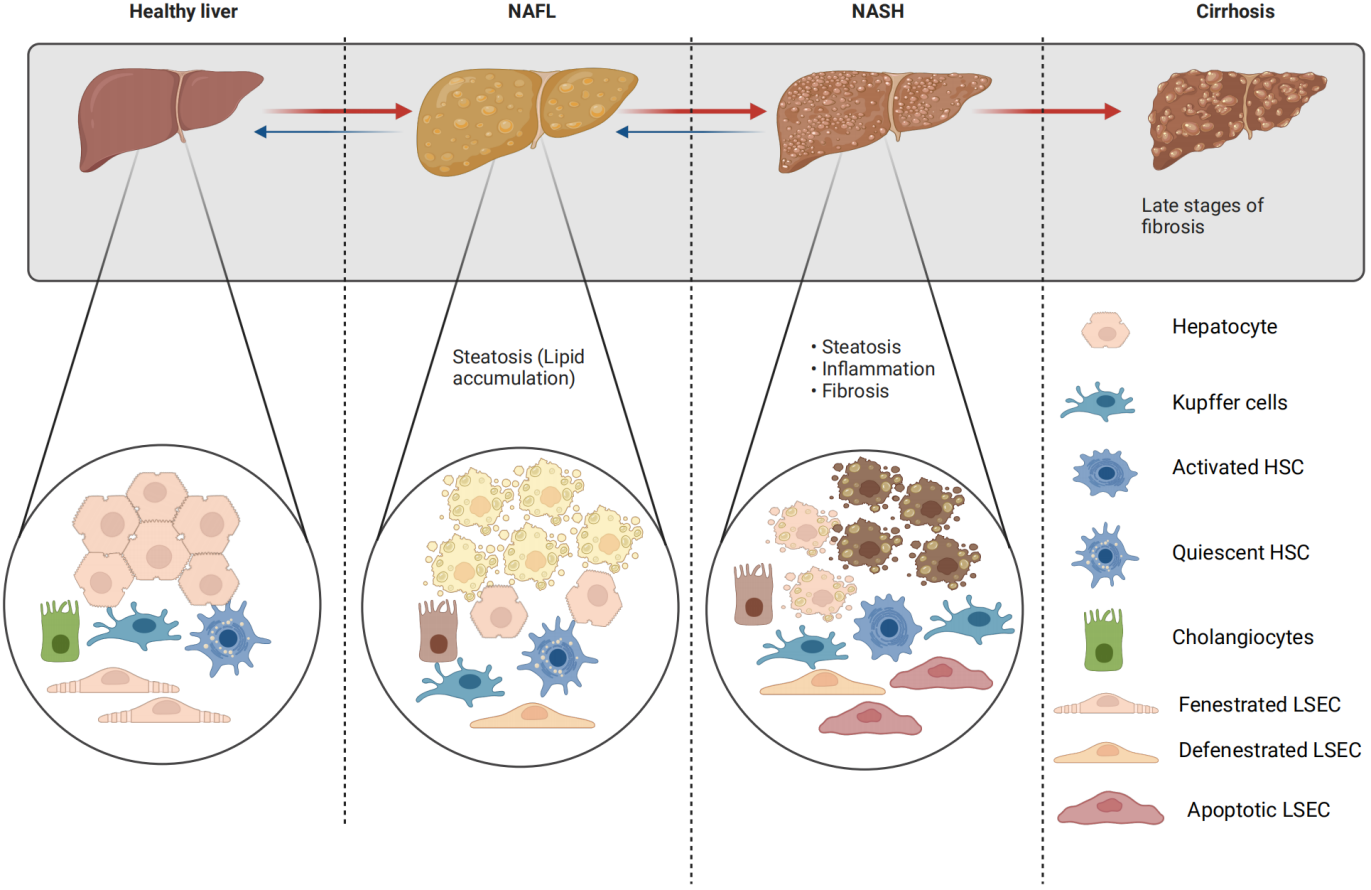
Changes of liver parenchyma and non-parenchymal cells in NAFLD. The transition between fenestrated and defenestrated LSECs: under physiological conditions, LSECs are perforated by fenestrations and lack a basement membrane; however, under pathological conditions, LSECs lose their fenestrations and form a continuous basement membrane. This phenomenon is called “capillarization”. Capillarization, i.e. loss of LSECs fenestrae, and LSECs dysfunction, i.e. the loss of the ability of LSECs to generate vasodilator agents in response to increased shear stress, are two events occurring early in NAFLD. The transition from quiescent to active HSCs: a distinct feature of quiescent hepatic stellate cells is the storage of retinoids (vitamin A and its metabolites) within their cytoplasmic lipid droplets. However, activated hepatic stellate cells exhibit a contractile, proliferative, and fibrogenic phenotype, which can be further distinguished from quiescent hepatic stellate cells by the loss of their retinol-containing lipid droplets.

In the pathological progression of NAFLD, various cell types including parenchymal and non-parenchymal hepatocytes, KCs, HSCs, liver sinusoidal endothelial cells (LSECs), and cholangiocytes are implicated (92) (**Figure 2**).

### 3.1 Hepatocytes in NAFLD

Under physiological conditions, hepatic lipid metabolism is typically mediated by fatty acid synthase (FAS), which facilitates the esterification of TG and very low-density lipoprotein (VLDL) for secretion into the circulation (93). Meanwhile, it can be oxidized in mitochondria through β-oxidation or stored as LDs, these LDs can then undergo lipolysis and fat phagocytosis to provide fatty acids for β-oxidation (94). In NAFLD, the increased lipolysis in the liver leads to the excessive release of FFA, which stimulates lipid accumulation and degeneration in hepatocytes, triggers mitochondrial dysfunction, activates ERS, and results in lipid peroxidation in the liver (8, 9). This process promotes excessive production of reactive ROS and damages liver autophagy. Furthermore, the generation of reactive ROS activates inflammatory signaling pathways, such as the nuclear factor κB (NF-κB) and JNK pathways, leading to an increase in inflammatory cytokines. As a result, this activation triggers KCs and accelerates the progression of NAFLD (94). Overexpression of miR-1297 in exosomes from lipotoxic hepatocytes can activate the PTEN/PI3K/AKT signaling pathway, promoting HSC activation and proliferation, inducing fibrosis, and accelerating the progression of NAFLD (95). Hepatocyte autophagy can enhance lipophagy, selectively degrade LDs, and thus improve lipid metabolism in NAFLD (96, 97). This topic will be further discussed in the subsequent section of the article.

### 3.2 Kupffer cells in NAFLD

KCs, the liver-resident macrophages, originate from yolk sac progenitor cells and are integral to liver homeostasis, inflammation, and fibrosis regulation (98, 99). The phagocytosis of apoptotic steatosis hepatocytes and uptake of free cholesterol are critical features of KCs in NAFLD. However, in NAFLD-damaged hepatocytes, resident KCs sense the disturbance of homeostasis and are activated. This activation stimulates the production of inflammation and the release of pro-inflammatory factors (IL-1β, IL-6, TNF-α) and pro-fibrotic factors, promoting the progression of NAFLD (11, 100). In addition, hepatic macrophages function by undergoing classical M1 activation or M2 activation (101). The regulation of NASH involves a delicate balance between pro-inflammatory M1 KCs and anti-inflammatory M2 KCs, with the activation of NLRP3 as a classical inflammasome in KCs (102). There is an increasing body of evidence suggesting that hepatic macrophages play a crucial role in the development of NAFLD (103–105). Studies have found that autophagy is an upstream regulator of KCs inflammasome polarization and activation. Increasing autophagy flux and inhibiting inflammasome activation are the main approaches to improving the inflammatory state of NAFLD by inhibiting M1 polarization and NLRP3 inflammasome activation (106).

### 3.3 Hepatic stellate cells in NAFLD

HSCs, situated in the Disse space between the lateral basal region of hepatocytes and the surface of sinusoidal endothelial cells, serve as the principal producers of collagen within the liver (107). In healthy livers, quiescent HSCs facilitate the intercellular transport of cytokines and soluble mediators (108). However, activation of HSCs enhances extracellular matrix (ECM) production, which is a key initiating event in hepatic fibrosis and plays a crucial role in NAFLD progression (109, 110). Specifically, factors such as inflammation, lipotoxicity, lipid mediators, and growth factors trigger hepatocyte death. A distinctive feature of HSCs is the storage of retinoids (vitamin A and its metabolites) within their cytoplasmic lipid droplets (111). Lipophagy occurs in HSCs, mediating the extensive β-oxidation of FFA, providing the necessary energy for HSC activation during the fibrosis process (66). Meanwhile, lipophagy promotes the decomposition of LDs and accelerates the activation of HSCs, which eventually leads to the progression of liver fibrosis (66). They also stimulate the recruitment of KCs, induce the production of transforming growth factor beta (TGF-β) and expression of alpha-smooth muscle actin (α-SMA), along with ECM synthesis. This leads to proliferation and activation of HSCs that transform into “myofibroblasts” (112). Activated HSCs contribute to inflammation and fibrosis in NAFLD by secreting proinflammatory cytokines and expressing adhesion molecules that promote the recruitment and infiltration of immune cells into the liver (113). The inhibition of the TGF-β pathway in a mouse model of NAFLD has been demonstrated to result in reduced activation of HSCs and alleviated fibrosis, with the most pronounced effect observed when IL13 is concurrently suppressed (114). However, the regulation of autophagy in HSCs is intricate, encompassing the activation of quiescent HSCs and the inhibition of activated HSCs. Recent studies have shown that activated HSCs are essential in the deposition of fibrotic tissue. However, autophagy inhibits the release of fibrogenic extracellular vesicles (EVs), thereby alleviating liver fibrosis (115). Furthermore, in mouse models of NAFLD, the activation of the PGE2/PGE2 receptor 4 (EP4) axis stimulates extracellular signal-regulated kinase pathways, enhances autophagy, and induces HSC activation and fibrosis (116).

### 3.4 Liver sinusoidal endothelial cells in NAFLD

LSECs are the predominant non-parenchymal cells in the liver and play a pivotal role in mediating the transport of nutrients, lipids, and lipoproteins (117). Under physiological conditions, LSECs are perforated by fenestrations and lack a basement membrane; however, under pathological conditions, LSECs lose their fenestrations and form a continuous basement membrane, this phenomenon is called ‘capillarization’, also known as defenestration, refers to the loss of fenestrae (118). LSECs facilitate bidirectional lipid exchange between the bloodstream and hepatic tissue (119). Additionally, they serve as a crucial defense barrier within the liver microenvironment by actively maintaining quiescence in HSCs and KCs (119). Conversely, the dysfunction of LSECs are an early event in the progression of NAFLD, leading to impaired hepatic lipid uptake and metabolism, inflammation, and hepatocyte damage (120–122). Specifically, impaired LSEC function reduces their permeability, resulting in formation of capillarization and hindering the release of VLDL from hepatocytes into the hepatic sinusoidal cavity (123). Consequently, cholesterol and TG accumulate in the liver, promoting the development of NAFLD. As this condition progresses, autophagy impairment, LSEC capillarization, inability to maintain KC dormancy, activation of the NF-κB pathway, reduction in endothelial nitric oxide synthase (eNOS), and increase in inducible nitric oxide synthase (iNOS) occur. Also, there is an upregulation of adhesion molecules and chemokines, all of which contribute to inflammation in patients with NAFLD (119, 124). Interestingly, capillarization LSECs also secrete fibrosis factors, such as TGF-β1 and extracellular matrix proteins, thereby stimulating the activation of adjacent HSCs and promoting the progression of NAFLD to liver fibrosis (125, 126). Studies have shown that nitric oxide in LSECs can temporarily activate KC while down-regulating proinflammatory chemokines involved in monocyte and macrophage recruitment through MAPK-dependent pathways (127). Hammoutene et al. (128) revealed LSEC apoptosis and inflammatory responses following a high-fat diet in LSEC-specific Atg5 knockout mice.

### 3.5 Cholangiocytes in NAFLD

The epithelium of intra- and extra-hepatic bile ducts is lined with cholangiocytes, which include two distinct types, small and large (129, 130). In the physiological state, cholangiocytes have a low proliferative capacity and are in a mitotic quiescent phase (131). In contrast, during the development of NAFLD, hepatocyte apoptosis leads to cholangiocyte injury (132). However, cholangiocytes participate in the progressive portal and bridging fibrosis of NAFLD through stimulation by fatty acids, injury caused by bile stasis, and influences on repair and proliferation. These factors promote the progression of NAFLD to liver fibrosis (133, 134). Specifically, damaged cholangiocytes rapidly activate into proliferating progenitor cells by releasing various cytokines, growth factors, neuropeptides, and hormones. This leads to manifestations such as proliferation promotion, inflammation promotion, fibrosis promotion, or aging (135, 136). Additionally, the expression and localization of bile acid receptors TGR5 and S1PR2 (sphingosine 1-phosphate receptor 2) in cholangiocytes are diminished, accompanied by a decrease in FXR activation (137, 138). This impairment in promoting downstream pathways of bile acids disrupts cholangiocyte bile acid homeostasis, elevating total bile acids and contributing to the NAFLD phenotype (137, 138). Research has shown that cholangiocytes promote hepatic steatosis by upregulating lipid biosynthesis genes by regulating the SCT/SCTR/miR-125b axis, which is crucial for improving the phenotype of NAFLD/NASH in humans (139).

## 4 NAFLD and autophagy

**Figure 3:**
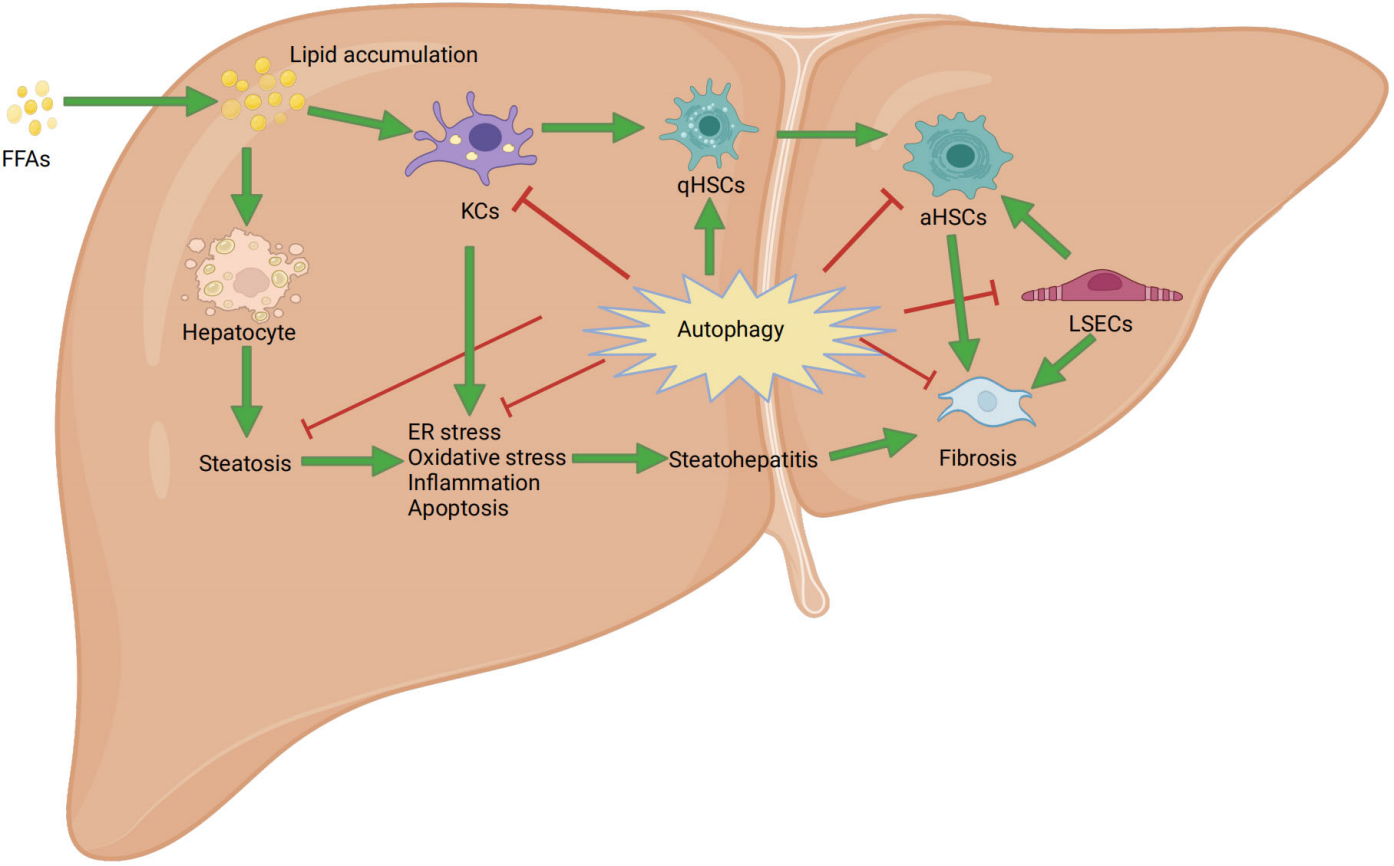
Mechanism of autophagy in nonalcoholic fatty liver disease. FFAs: free fatty acids; ER: endoplasmic reticulum stress; aHSCs: activated hepatic stellate cells; qHSCs: Quiescent hepatic stellate cells; KCs: Kupffer cells; LSECs: liver sinusoidal endothelial cells.

As previously described, we discussed the biological processes and degradation of autophagy, as well as its function in cellular metabolism. Through a literature review, we demonstrated the close association between autophagy and the progression of NAFLD in both liver parenchyma and non-parenchymal cells (**Figure 3**). However, there is a lack of research on the treatment of NAFLD by regulating autophagy through drug-targeting signaling pathways for LSECs and cholangiocytes. Therefore, we will start by focusing on the formation of autophagosomes and autolysosomes in three specific cell types, namely hepatocytes, KCs, and HSCs. This will allow us to explore how drugs targeting autophagy through signaling pathways can be used to treat the progression of NAFLD, thereby deepening our understanding of the relationship between autophagy and NAFLD.

### 4.1 The role of autophagosome formation in NAFLD

#### 4.1.1 The role of autophagy in hepatocytes

**Figure 4:**
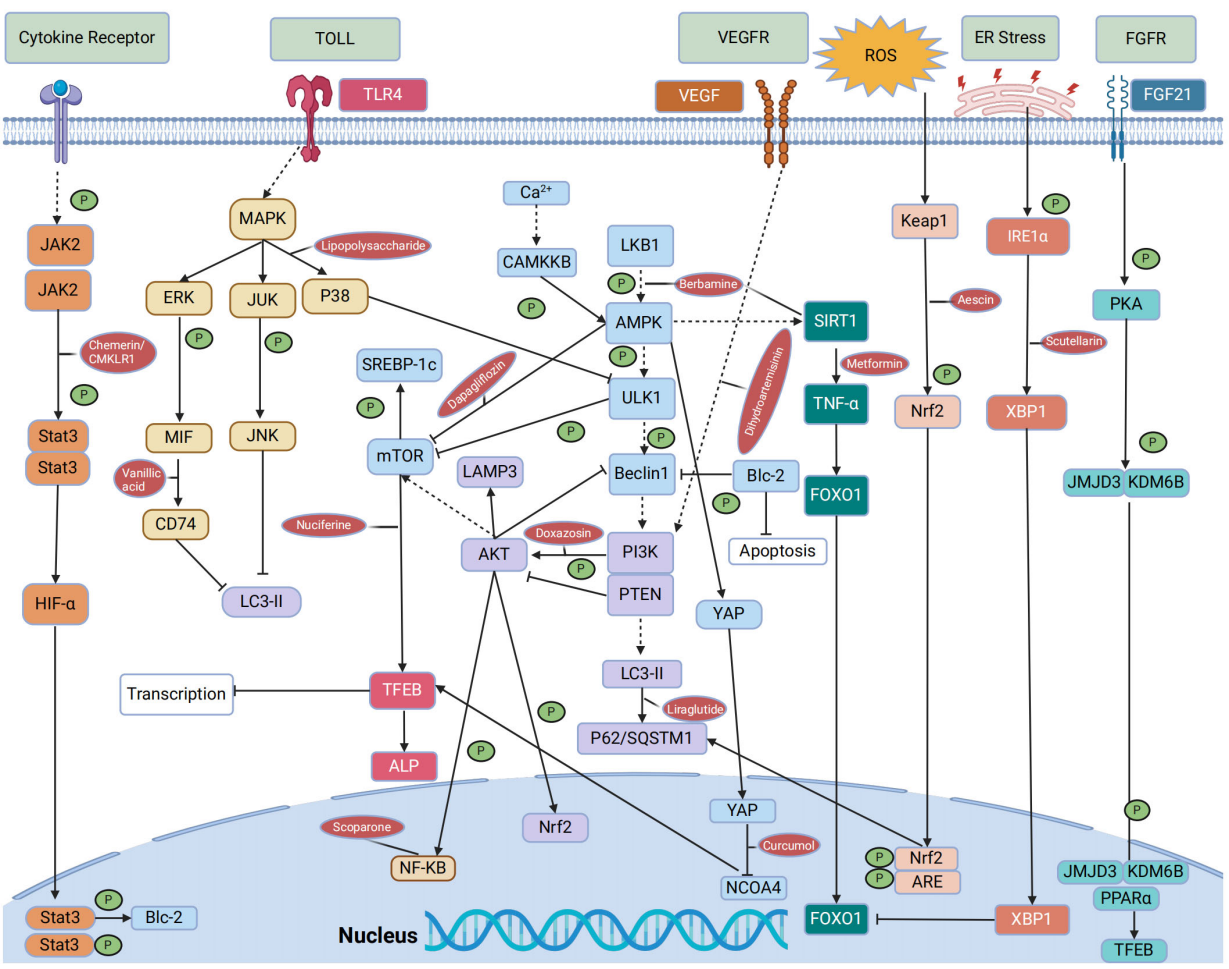
Signaling pathways that regulate autophagy or are regulated by autophagy in NAFLD.

In mouse models of NAFLD, pharmacological agents have been shown to modulate autophagy via key metabolic pathways, thus influencing the disease’s progression. Compounds such as schisandrin B and acetylshikonin (AS) activate the AMPK/mTOR pathway, stimulating hepatocyte autophagy and attenuating NAFLD progression (140, 141). Similarly, empagliflozin activates the AMPK/mTOR pathway, upregulates LC3B expression, induces autophagy, and improves NAFLD in apolipoprotein E (ApoE) knockout mouse models (142). In the ob/ob mouse model of NAFLD, metformin enhances the activity of PRKA by activating SIRT1-FOXO signaling pathways, thereby inducing autophagy and facilitating lipid metabolism in NAFLD (143). Berbamine (BBM) similarly invokes the SIRT1/LKB1/AMPK pathway to enhance autophagy markers and induce autophagy, slowing down NAFLD progression (144). However, in a HepG2 model induced by palmitic acid (PA), dapagliflozin, an SGLT2 inhibitor, induces fatty acid oxidation and autophagy through the AMPK-mTOR-ULK1 pathway (145). Pueraria radix flavonoids stimulate autophagy by inhibiting the PI3K/Akt/mTOR signaling pathway, reducing intracellular lipid accumulation and inflammation, while ginsenoside Rg1 modulates the PTEN-AKT pathway to mitigate NAFLD progress (146, 147). Dual inhibitions of soluble epoxide hydrolase (sEH) and cyclooxygenase-2 (COX-2), along with the GLP-1 analog liraglutide (LRG), activate the Sirt1/PI3K/AKT/mTOR and AMPK/mTOR/Beclin1 pathways to promote autophagy, alleviating NAFLD (148–150). Ursodeoxycholic acid (UDCA) modulates the Bcl-2/Beclin-1 and Bcl-2/Bax complexes via an AMPK pathway, inducing autophagy and impeding the progression of NAFLD (151). Conversely, acetaminophen (APAP) overdose reduces LC3-II, Beclin1, and AMPK levels while increasing mTOR and SREBP-1c levels, this inhibits autophagy and exacerbates NAFLD lipid accumulation (152). Chemerin/CMKLR1 enhances autophagy and reduces hepatic oxidative stress via the JAK2-STAT3 pathway (153). Interestingly, fasting-induced fibroblast growth factor-21 (FGF21) signaling activates PKA, which phosphorylates JMJD3, enhancing its nuclear localization and interaction with the nuclear receptor PPARα, this promotes hepatic autophagy and lipid degradation in hepatocytes (154). In another experimental study, aescinate activated the Keap1-Nrf2 pathway and autophagy, thereby ameliorating the progression of NAFLD (155). Compounds like magnolol (MG), in HepG2 and Wistar rat models, demonstrate therapeutic potential by inhibiting mTOR and activating the Nrf2-ARE pathway, enhancing autophagic flux and reversing hepatic steatosis (156). Additional studies with curcumin, 1,3-dichloro-2-propanol, icariin, baicalein, and verapamil have elucidated its effects on autophagic processes in hepatocyte models of NAFLD (157–161). Thus, autophagy regulates hepatocyte metabolism through mTOR, AMPK, PI3K, AKT, and PPAR pathways. These pathways can mitigate steatosis, ameliorate oxidative stress and liver injury, and slow the progression of NAFLD (**Figure 4**, **Tables 1**–**3**).

**Table 1:** The role of autophagosome formation in NAFLD (drugs with clinical trials).

Treatment	Signaling pathway	Modeling method (animal/cell)	Conclusion	References
Berbamine(BBM)	SIRT1/LKB1/AMPK signaling pathway	Wistar rats	BBM mitigates liver lipid metabolism disorders by modulating the SIRT1/LKB1/AMPK pathway, regulating the expression of autophagy markers LC3a/b, Beclin 1, and p62, and inducing autophagy to decelerate the progression of NAFLD.	(144)
Dapagliflozin	AMPK-mTOR signaling pathway	ZDF rats and ZL rats; PA-induced LO2 and HepG2 cells	Dapagliflozin affects hepatic steatosis in NAFLD by downregulating lipogenic enzymes and activating the AMPK-mTOR pathway, while promoting fatty acid oxidation and autophagy to mitigate disease progression.	(145)
Doxazosin	PI3K/Akt/mTOR signaling pathway	CCl4 induced C57BL/6J mice;LX-2 cells	Doxazosin inhibits autophagy by activating the PI3K/Akt/mTOR signaling pathway, attenuating liver fibrosis.	(162)
Empagliflozin	AMPK/mTOR signaling pathway	HFD-fed ApoE(-/-) mice	Empagliflozin activates the AMPK/mTOR pathway, upregulates LC3B expression, induces autophagy, and ameliorates NAFLD	(142)
Empagliflozin	AMPK/mTOR signaling pathway	HFD induced C57BL/6J mice	Empagliflozin significantly enhances the autophagy of liver macrophages through the AMPK/mTOR signaling pathway, inhibits the expression level of IL-17/IL-23 axis-related molecules, reduces inflammatory response, and improves NAFLD-related liver injury.	(163)
Imatinib	STAT3/IL-6 signaling pathway	LX-2 cell;CCl4 induced SD rats	Imatinib suppresses the activation of HSCs through the specific targeting of the STAT3/IL-6 pathway via miR-124.	(164)
Liraglutide (LRG)	AMPK/mTOR/Beclin1 signaling pathway	HFD induced C57BL/6 mice;FFA induced L-O2 cells	LRG induces autophagy through the AMPK/mTOR/Beclin1 pathway, regulating the expression of autophagy proteins SQSTM1/P62 and LC3B, thereby improving hepatic lipid accumulation in NAFLD.	(149)
Metformin	PRKA/SIRT1/FOXO signaling pathway	ob/ob mice	Metformin targets PRKA to activate the SIRT1/FOXO signaling pathway, thereby inducing autophagy and slowing down the progression of NAFLD.	(143)
Metformin	AMPK-SIRT1 signaling pathway	Ttp mice;KCs; PHH	Metformin activates TTP through the AMPK-Sirt1 pathway, inhibits the production of KCs to suppress TNF-α, downregulates Rheb expression, inhibits mTORC1 expression, enhances TFEB nuclear translocation, and promotes autophagy.	(165)
Scoparone	ROS/P38/Nrf2 axis and PI3K/AKT/mTOR signaling pathway	MCD induced C57BL/6;LPS induced RAW264.7 and PA-induced AML12 cells	Scoparone can ameliorate liver inflammation and enhance autophagy in NASH mice by inhibiting the ROS/P38/Nrf2 axis and PI3K/AKT/mTOR pathway, thereby promoting autophagic flux while suppressing inflammation.	(166)
Scoparone	TLR4/NF-κB signaling pathway	MCD induced C57BL/6 mice;RAW264.7 cells	Scoparone mitigates inflammation, apoptosis, and fibrosis in NASH by suppressing mice’s TLR4/NF-κB signaling pathway.	(167)
Ursodeoxycholic acid (UDCA)	Beclin-1-Bcl-2 complex-mediated signaling pathway	HFD induced SD rats	UDCA modulates the interaction between the Bcl-2/Beclin-1 complex and the Bcl-2/Bax complex through activation of the AMPK pathway, thereby inducing autophagy and impeding the progression of NAFLD.	(151)
Verapamil	mTOR signaling pathway	HFD indeced C57BL/6 mice	Verapamil induces autophagy through an mTOR-independent signaling pathway, improves hepatocyte function, and promotes hepatocyte regeneration.	(161)

NAFLD, non-alcoholic fatty liver disease; SIRT1, sirtuin1; LKB1, liver kinase B1; AMPK, AMP-activated protein kinase; mTOR, mammalian target of rapamycin; ZDF rats, Zucker Diabetic Fatty rats; ZL rats, Zucker lean rats; PA, palmitic acid; LO2, human normal liver cell; HepG2, human hepatocellular carcinomas; CCl4, carbon tetrachloride; LX-2 cells, human hepatic stellate cells; PI3K, phosphatidylinositol three kinase; AKT, protein kinase B; HFD, high fiber diet; CCl4, carbon tetrachloride; SD, Sprague-Dawley rats; FFA, free fatty acid; IL-17, interleukin 17; IL-23, interleukin,23; STAT3, signal transducer and activator of transcription 3; IL-6, interleukin 6; PRKA, AMP-activated protein kinase; FOXO, forkhead box O; ob/ob mice, obese mice; PHH, primary hepatocyte model; TTP, tristetraprolin; TNF-α, tumor necrosis factor-α; KCs, kupffter cells; TFEB, transcription factor EB; Nrf2, Nuclear factor erythroid2-related factor 2; MCD, methionne-choline deficient diet; LPS, lipopolysaccharide; RAW 264.7 cells, mouse mononuclear macrophages cells; AML12 cells, alpha mouse liver 12 cells; TLR4, toll-like receptor 4; NF-κB, nuclear factor kappa-B;NASH, nonalcoholic steatohepatitis.

**Table 2:** The role of autophagosome formation in NAFLD (drugs without clinical trials).

Treatment	Signaling pathway	Modeling method (animal/cell)	Conclusion	References
Acetylshikonin (AS)	AMPK/mTOR signaling pathway	MCD induced C57BL/6 mice	AS enhances hepatocyte autophagy via the AMPK/mTOR pathway to ameliorate NAFLD.	(141)
Aescin (Aes)	Keap1-Nrf2 signaling pathway	Nrf2/C57BL/6 mice;Atg5/C57BL/6 mice	Aescin effectively alleviated NAFLD by regulating the Keap1-Nrf2 pathway and activating antioxidant mechanisms and autophagy	(155)
Alisol A 24-acetate	AMPK/mTOR/ULK1 signaling pathway	LX-2 cells;MCD induced C57BL/6 mice	Alisol A 24-acetate exerts its therapeutic effects on NASH by activating autophagy via the AMPK/mTOR/ULK1 pathway, suppressing the expression of pro-inflammatory cytokines and reactive ROS, and alleviating oxidative stress.	(168)
Curcumol	YAP/NCOA4 axis	C57BL/6J mice; LO2 cells	Curcumol inhibits hepatocyte senescence by regulating NAFLD iron autophagy through YAP/NCOA4.	(157)
Curcumin derivative	AMPK/TGF-β signaling pathway	TGF-β induces LX-2 cells and hepatocytes (alpha mouse liver 12 [AML12])	Curcumin derivatives combined with TGF-β receptor I inhibitors can attenuate liver fibrosis and impede the progression of NAFLD.	(169)
Curcumin	PI3K/Akt/mTOR signaling pathway	HSCs line LX-2 cells	The activation of the PI3K/Akt/mTOR signaling pathway by curcumin can effectively inhibit autophagy, suppress the activity, and induce apoptosis in LX-2 cells derived from the HSCs line, thereby attenuating the progression of liver fibrosis.	(170)
Dihydroartemisinin	VEGF/PI3K/AKT/mTOR/ULK1 signaling pathway	SD rats;HSC−LX2 cells	The regulation of autophagy by dihydroartemisinin involves the modulation of the VEGF pathway and mediation of the PI3K/AKT/mTOR/ULK1 pathway, leading to inhibition of HSCs activation and delayed progression of NAFLD.	(171)
Ginsenoside Rg1	PTEN-AKT signaling pathway	FFA induced HepG2;MCD induced C57BL/6 mice	Ginsenoside Rg1 exerts its effects on autophagy and pyroptosis by modulating the miR-375–3p/ATG2B/PTEN-AKT pathway, thereby mitigating the pathogenesis and progression of NAFLD.	(147)
Glycyrrhetinic acid	STAT3-HIF-1α signaling pathway	C57BL/6 mice;KCs cells	Glycyrrhetinic acid modulates the STAT3-HIF-1α pathway in macrophages, enhancing autophagy flux impairment. This mitigates the excessive generation of inflammatory cytokines and hepatocyte apoptosis, thereby alleviating the progression of NAFLD.	(172)
Icaritin	AMPK signaling pathway	sodium oleate-induced L02 and Huh-7 cells	Icaritin attenuates lipid accumulation by increasing energy expenditure and autophagy regulated by phosphorylating AMPK.	(159)
Lipopolysaccharide(LPS)	MAPK p38/Ulk1 signaling pathway	LPS-induced HSC-T6 cells	The expression of IL-1β induced by autophagy in HSCs is inhibited by regulating the MAPK p38/Ulk1 pathway in LPS-induced HSC-T6 cells.	(173)
Magnolol(MG)	Nrf2-ARE and mTOR signaling pathway	PA-induced HepG2; Tyloxapo-induced Wistar rats	MG inhibits mTOR and activates the Nrf2-ARE pathway, enhancing autophagic flux and ameliorating hepatocyte steatosis.	(156)
Pueraria flavonoids	PI3K/Akt/mTOR signaling pathway	C57BL/6J mice were induced by 40% fat; PA-induced HepG2 cells	Pueraria radix flavonoids induce autophagy by inhibiting the PI3K/Akt/mTOR signaling pathway, thereby reducing intracellular lipid accumulation and inflammation levels, ultimately ameliorating NAFLD.	(146)
Palmitic acid	Hh signaling	LX2、HSCs and rat BSC-C10 cells	The activation of HSCs is induced by palmitic acid through the inflammasome and Hh signaling pathways.	(174)
Resveratrol	SIRT1 and JNK signaling pathway	Immortalized mouse HSC line JS1 cells	Resveratrol modulates autophagy and apoptosis via the SIRT1 and JNK signaling pathways, suppresses HSCs activation, and attenuates liver fibrosis in NAFLD.	(175)
Schisandrin B(Sch B)	AMPK/mTOR signaling pathway	FFA induced HepG2;HFD induced C57BL/6J mice	Sch B activates autophagy through the AMPK/mTOR pathway, inhibits steatosis, and promotes fatty acid oxidation, thereby alleviating the progression of NAFLD.	(140)
Soluble epoxide hydrolase(sEH)/cyclooxygenase-2(COX-2) Dual Inhibitor	Sirt1/PI3K/AKT/mTOR signaling pathway	HFD induced C57BL/6J mouse;PA-induced AML12 hepatocytes	The COX-2/EH complex can suppress the PI3K/AKT/mTOR signaling pathway via Sirt1, enhancing autophagy, decelerating hepatocyte senescence, and ameliorating NAFLD.	(148)
Scutellarin (Scu)	IRE1α/XBP1/FoxO1 signaling pathway	PA-induced HepG2 cells;HFD induced C57/BL6 mice	Scu down-regulates SREBP-1c expression through the IRE1α/XBP1/FoxO1 pathway, inhibits endoplasmic reticulum stress, and enhances autophagy to improve liver lipid accumulation.	(160)
Salvianolic acid B	MAPK/p38/JUK signaling pathway	JS1 and LX2 cells	The MAPK pathway is down-regulated by salvianolic acid B to inhibit TGF-β1-induced autophagy and activation of HSCs.	(176)
Vanillic acid	MIF/CD74 signaling pathway	ccl4 induced SD rats;HSCs-T6cells	The progression of NAFLD to liver fibrosis can be alleviated by vanillic acid, which inhibits the autophagy of HSCs through the MIF/CD74 signaling pathway.	(177)
1,3-dichloro-2-propanol (1,3-DCP)	AKT/mTOR/FOXO1 signaling pathway	C57BL/6 mice; HepG2 cells	1, 3-DCP activates the phosphorylation of AKT and mTOR, reduces the expression of FOXO1, and inhibits autophagy-mediated lipid accumulation	(158)

Keap1, kelch-like ECH-associated protein-1; ULK1, unc-51-like kinase 1; YAP, Yes-associated protein; NCOA4, nuclear receptor coactivator 4; TGF-β, Transforming growth factor beta; HSCs, hepatic stellate cells; VEGF, vascular endothelial-derived growth factor; PTEN, phosphatase and tensin homolog; HIF-1α, hypoxia-inducible factor-1α; MAPK, mitogen-activated protein kinase; ARE, anti-oxidative response element; Hh, Hedgehog; JS1, the mouse immortalized stellate cell lines; JNK, Jun N-terminal kinase; IRE1α/XBP1, inositol-requiring enzyme 1α(IRE1α)/X-box-binding protein 1 (XBP1); FOXO1, Forkhead box O1; MIF, migration inhibitory factor;

**Table 3:** The role of autophagosome formation in NAFLD (low or high expression of a particular target).

Treatment	Signaling pathway	Modeling method (animal/cell)	Conclusion	References
Acetaminophen (APAP)	AMPK/mTOR/SREBP-1c signaling pathway	C57BL/6J mice;L02 cells	The overdose of APAP reduces LC3-II and Beclin1 levels through the AMPK/mTOR/SREBP-1c pathway, inhibiting autophagy and exacerbating the progression of NAFLD.	(152)
Angiotensin-converting enzyme 2 (ACE2) overexpression	AMPK/mTOR signaling pathway	rAAV2/8-ACE2 induced C57 mice;	Overexpression of the ACE2 protein activates the AMPK/mTOR pathway, modulates HSCs autophagy, suppresses HSCs activation, and decelerates the progression of liver fibrosis in individuals with NAFLD.	(178)
Chemerin/CMKLR1	JAK2-STAT3 signaling pathway	HFD-induced C57BL/6 mice	The regulation of the JAK2-STAT3 pathway by Chemerin/CMKLR1 can augment autophagy and ameliorate hepatic oxidative stress, thereby enhancing NASH.	(153)
Jumonji-D3 (JMJD3/KDM6B)	PPARα signaling pathway	HFD induced JMJD3-floxed mice;Hepa1c1c7 cells	FGF21 signaling triggers the PPARα pathway through JMJD3/KDM6B histone demethylase, promoting hepatic autophagy and hepatocyte lipid degradation in NAFLD.	(154)
Lp-PLA2 low expression	JAK2/STAT3 signaling pathway	HFD induced C57BL/6J mice;KCs	The down-regulation of Lp-PLA2 expression inhibits the JAK2/STAT3 signaling pathway, reduces lipid accumulation, promotes autophagy, suppresses the production of inflammatory factors, and delays the progression of NAFLD.	(179)
Maresin 1	AMPK-SERCA2b signaling pathway	HFD-induced C57 mice	Maresin 1 mitigates NAFLD by attenuating endoplasmic reticulum stress via the AMPK-SERCA2b pathway.	(180)
TIM-4	Akt4/Mitophagy signaling pathway	C57 mice; KCs	The inhibition of ccl4-induced liver fibrosis by TIM-4 is achieved through interference with KCs via the Akt4/mitophagic signaling pathway, and it also delays the progression of NAFLD.	(181)
Tim-4	LKB3/AMPKα signaling pathway	C57BL/6 mice; KCs and HSCs	The inhibition of the NLRP1 inflammasome by Tim-4 in liver macrophages through the LKB3/AMPKα pathway serves as an intervention strategy for NAFLD progression.	(182)

rAAV2/8-ACE2,recombinant adeno-associated virus ACE2 vector; JAK2-STAT3, the Janus kinase (JAK)2/signal transductors and the transcription (STAT)3; Chemerin/CMKLR1, chemerin chemokine-like receptor 1 Gene; PPARα, peroxisome proliferator-activated receptor-α; FGF21, fibroblast growth factor; JMJD3,Jumonji domain-containing protein D3; Hepa1c1c7 cells, mouse hepatocarcinoma cells.

#### 4.1.2 The role of autophagy in Kupffer cells

Scoparone, for instance, has been shown to enhance autophagic flux and suppress inflammation by inhibiting the ROS/P38/Nrf2 axis and the PI3K/AKT/mTOR pathway in an LPS-induced liver macrophage model (166). It also attenuates inflammation, apoptosis, and fibrosis progression in NAFLD through the downregulation of the TLR4/NF-κB signaling pathway (167). Furthermore, silencing lipoprotein-associated phospholipase A2 (Lp-PLA2) in KCs and NAFLD model mice inhibited the activation of the JAK2/STAT3 signaling pathway, mitigated lipid accumulation, promoted autophagy, reduced production of inflammatory factors, and inhibited the progression of NAFLD (179). In Ttp mice models of KCs and primary hepatocytes, metformin activates the mRNA-binding protein tristetraproline (TTP) through the AMPK-Sirt1 pathway in both hepatocytes and KCs, this activation curtails TNF-α production in KCs, downregulates Rheb expression, suppresses mTORC1 expression, and enhances TFEB nuclear translocation (165). Collectively, these effects promote autophagy and alleviate NAFLD progression. In addition, the presence of TIM-4 on KCs acts as a negative regulator, reinstating autophagy through the Akt4/mitophagy signaling pathway and modulating the progression of NAFLD by exerting inhibitory effects on the NLRP1 inflammasome via the LKB3/AMPKα pathway (181, 182). Glycyrrhizic acid has been demonstrated to enhance impaired autophagy flux, mitigate excessive production of inflammatory cytokines, and ameliorate hepatocyte apoptosis through the regulation of the STAT3-HIF-1α pathway in macrophages (172). Empagliflozin has also been reported to significantly enhance autophagy in hepatic macrophages through the AMPK/mTOR pathway, suppressing the IL-17/IL-23 axis and improving liver injury related to NAFLD (163). Moreover, maresin 1, a twenty-two carbon polyunsaturated fatty acid synthesized by macrophages, reinstates autophagy and suppresses ER via the AMPK-SERCA2b pathway (180). In summary, autophagy plays a crucial role in maintaining the homeostasis and functionality of KCs. Autophagy in hepatic macrophages can modulate the polarization and activation of inflammasomes, thereby mitigating the progression of NAFLD through its antioxidant and anti-inflammatory effects. Conversely, impaired autophagy can worsen inflammatory responses in KCs, induce ERS, and expedite the onset and progression of diseases. This detrimental cascade can be mitigated by pharmacological agents such as metformin, scoparone, and empagliflozin, which modulate autophagy-related pathways (**Figure 4**; **Tables 1**–**3**).

#### 4.1.3 The role of autophagy in hepatic stellate cells

Resveratrol has been shown to induce autophagy in immortalized mouse HSCs, dampening HSC activation through the SIRT1 and JNK signaling pathways, thus attenuating liver fibrosis (175). Salvianolic acid B, by influencing the MAPK pathway, has been effective in suppressing TGF-β1-induced autophagy and HSC activation, as well as modulating ERK, p38, and JNK signaling cascades (176). In both CCl4-induced SD rat and HSC models, vanillic acid treatment was found to inhibit the MIF/CD74 signaling pathway, subsequently suppressing autophagy (177). When curcumin derivatives are combined with a TGF-β receptor I inhibitor in LX-2 liver fibrosis models, they activate the AMPK/TGF-β signaling pathway, regulate autophagy, and alleviate liver fibrosis (169). Curcumin itself activates the PI3K/Akt/mTOR signaling pathway, inhibiting autophagy and promoting apoptosis in LX-2 cells (170). Alisol A 24-acetate induces autophagy via the AMPK/mTOR/ULK1 pathway in MCD mice and LX-2 models (168). However, the activation of the VEGF pathway by dihydroartemisinin, along with its regulation of the PI3K/AKT/mTOR/ULK1 pathway, facilitates autophagy and retards the progression of NAFLD (171). Doxazosin has been observed to inhibit autophagy by stimulating the PI3K/Akt/mTOR pathway, attenuating liver fibrosis (162). Overexpression of angiotensin-converting enzyme 2 (ACE2) modulates autophagy via the AMPK/mTOR pathway in HSCs, suppressing HSC activation and promoting apoptosis (178). In the mouse model of HSCs, imatinib specifically targeted the STAT3/IL-6 pathway via miR-124, leading to the induction of autophagy and suppression of HSC activation (164). Conversely, PA induces HSC activation through the NLRP3 inflammasome and Hedgehog (Hh) signaling pathways, diminishing autophagic flux, inhibiting autophagy, and promoting the progression of NASH toward fibrosis (174). Additionally, in LPS-induced HSC-T6 cells, activation of the MAPK p38/Ulk1 pathway inhibits autophagy in HSCs and induces IL-1β expression (173). Based on the literature above, it has been observed that autophagy plays a dual role in HSCs. Dysfunction of autophagy facilitates energy supply for HSC activation during the development of fibrosis and contributes to the progression of NAFLD. On the other hand, autophagy also plays a role as an anti-fibrotic factor. It may promote HSC death, mediate the degradation of profibrotic mediators such as collagens and metalloproteinases, or diminish the release of exosomes that carry pro-fibrotic signals. This decelerates the progression of NAFLD to liver fibrosis. However, modulation of autophagy-related pathways through drug administration, such as resveratrol, dihydroartemisinin, and doxazosin, can enhance autophagic processes (**Figure 4**; **Tables 1**–**3**).

### 4.2 The role of autolysosome in NAFLD

In a NAFLD mouse model and a PA-induced HepG2 cell model, liraglutide has been observed to alleviate hepatic steatosis by modulating the expression of autophagy substrates LC3-II and SQSTM1/P62, thereby activating the autophagy-lysosomal pathway (ALP) regulated through the TFEB pathway (183). The PPARα agonist fenofibrate activates lysosomal Ca^2+^ via mucolipin 1, leading to stimulates the calcineurin phosphatase and CaMKKβ-AMPK-ULK1 signaling pathways (184). These pathways trigger TFEB activation and enhance lipophagy, which ameliorates fat accumulation in NAFLD. Nuciferine has been shown to effectively regulate the mTORC1-TFEB-ALP axis, suppressing the lysosomal localization and activity of mTORC1, activating TFEB-mediated ALP, and restoring autophagy (185). Simultaneously, quercetin enhances the co-localization of lysosomes and LDs through the IRE1a/XBP1s pathway while reducing their accumulation. It also stimulates autophagy to ameliorate HFD-induced NAFLD (186). Phillygenin reduces hepatocyte lipid deposition by regulating the Ca^2+^ calcineurin-TFEB axis, thereby enhancing lysosomal biogenesis and autophagic flux (187). The upregulation of lysosome-associated membrane protein 3 (LAMP3) in patients with NAFLD and in mouse models has been correlated with the activation of the PI3K/Akt pathway, which facilitates autolysosome fusion (188). Additionally, in HepG2 cells and HFD-induced C57BL/6J mice, the administration of lipid-lowering granules and activation of the PI3K-AKT-mTOR signaling pathway resulted in the induction of autophagosomes or their co-localization with lysosomes, this led to autophagosomal degradation, activation of the autophagy process, reduction in oxidative stress levels, and improvement in lipid accumulation and inflammatory response (189). Baicalein has been found to inhibit the mTOR signaling pathway, enhance lysosomal membrane permeability, and restore autophagy, offering a therapeutic strategy for NAFLD (190). Ajugol, an active alkaloid derived from the root of rehmanniobium, effectively inhibits mTOR and induces the nuclear translocation of TFEB, promoting the restoration of the autophagy-lysosomal pathway and lipophagy (191). In conclusion, the formation of autolysosomes is another key pathway during autophagy related to the degradation of intracellular metabolites (192). The dysregulation of the vital autophagy factor TFEB impaired lysosomal membrane activity and lysosomal biogenesis, leading to impaired lysosomal function and disruption of the autophagic degradation pathway (193). However, treatments such as liraglutide and quercetin have shown potential in restoring lysosomal function by promoting autophagy and decelerating the progression of NAFLD (**Figure 4**; **Table 4**).

**Table 4:** The role of autolysosome formation in NAFLD.

Treatment	Signaling pathway	Modeling method (animal/cell)	Conclusion	References
Ajugol	mTOR/TFEB signaling pathway	PA-induced hepatocytes;HFD induced C57BL/6J mice;	Ajugol inhibits mammalian targets of mTOR and induces nuclear translocation of TFEB, thereby promoting the TFEB-mediated autophagy-lysosomal pathway and lipo autophagy to ameliorate NAFLD.	(191)
Baicalein	mTOR signaling pathway	PA-induced HepG2;HFD induced C57BL/6J mice;	Baicalein can inhibit the mTOR signaling pathway and improve lysosomal membrane permeability in treating NAFLD.	(190)
Fenofibrate	CaMKKβ-AMPK-ULK1 signaling pathway	HFD induced mice;HepG2	The CaMKKβ-AMPK-ULK1 signaling pathway is regulated by fenofibrate, leading to the activation of lysosomal Ca^2+^ levels, induction of TFEB activation, promotion of fat phagocytosis, and ultimately improvement in fat accumulation in NAFLD.	(184)
Jiang Zhi Granule	PI3K-AKT-mTOR signaling pathway	PA induced HepG2;HFD induced C57BL/6J mice;	Jiangzhi granule induces either autophagosome formation or the co-localization of autophagosomes and lysosomes through the PI3K-AKT-mTOR signaling pathway, facilitating the degradation of autophagosomes to prevent hepatocyte injury effectively.	(189)
Liraglutide	TFEB-mediated signaling pathway	HFD induced C57BL/6J mic e; HepG2	Liraglutide ameliorates hepatic steatosis by inducing activation of the TFEB-mediated ALP through modulation of LC3-II and SQSTM1/P62 autophagy substrates expression levels.	(183)
Lysosome-associated membrane protein 3 (LAMP3)	PI3K/Akt signaling pathway	FFA induced HCC cells;HFD induced C57BL/6J mice;ob/ob mice	The overexpression of LAMP3 induces activation of the PI3K/Akt pathway, reducing TG content and promoting autolysosome fusion.	(188)
Nuciferine	mTORC1-TFEB-ALP signaling pathway	HFD induced C57BL/6J mice; HepG2	Nuciferine modulates hepatic steatosis and insulin resistance via the mTORC1-TFEB-ALP axis to attenuate the progression of NAFLD.	(185)
Phillygenin	Ca^2+^calcineurin-TFEB axis	PA-induced AML12 cells and primary hepatocytes;HFD induced C57BL/6J mice;	Phillygenin exerts a regulatory effect on the Ca^2+^ calcineurin-TFEB axis, enhancing lysosomal biogenesis and autophagy flux in hepatocytes and reducing lipid deposition.	(187)
Quercetin	IRE1a/XBP1s signaling pathway	HFD-induced SD rats; FFA-induced HepG2 cells	The activation of IRE1a/XBP1s by quercetin effectively enhances the colocalization of lysosomes with lipid droplets, reduces p62 accumulation, and promotes liver fat autophagy in NAFLD.	(186)

ALP, autophagy-lysosomal pathway; CaMKKβ, calcium/calmodulin-dependent protein kinase kinase β protein.

## 5 Discussion

Defects in autophagy are implicated in the pathogenesis of numerous diseases, including tumors, neurodegenerative disorders, and metabolic conditions (16). Moreover, dysfunctional autophagy is intricately associated with the pathological progression of NAFLD (194).

The upstream proteins activate AMPK in autophagy to facilitate phosphorylation of the ULK-1 complex and suppress activation of mTOR (29, 30). Subsequently, phosphorylation of downstream PI3KC3 complex I induces recruitment of ATG9, a downstream autophagy factor, onto the cell membrane and initiates autophagosome nucleation. Through interaction between DFCP1 and WIPI proteins, PI3P is generated, which facilitates cell nucleation and autophagosome formation (33, 34). Concurrently, the induction of WIPI and ATG16L1 triggers the formation of two downstream ubiquitin complexes: ATG12-ATG5-ATG16L1 and LC3-II (33, 35, 37, 38). These complexes are crucial in facilitating autophagosome elongation. Subsequently, the ubiquitination of LC3II through its association with p62/SQSTM1 as an autophagosomal substrate facilitates autophagosome maturation (43). In conjunction with the HOPS complex, VPS34 complex II and Rab7 further regulate this process, enabling fusion between cargo-containing autophagosome sand lysosomes (45, 47, 48). Ultimately, lysosomes digest this fused product, leading to degradation, reactivating mTORC1, inhibiting autophagy, and terminating the autophagic process (51). The degradation modes within autophagy encompass both non-selective and selective processes. Non-selective autophagy is a cellular process that eliminates senescent or dysfunctional components like organelles and proteins due to nutrient deficiency or stress conditions while degrading them for recycling. This process helps maintain cellular energy balance (54). On the other hand, selective autophagy is a cellular response mechanism that targets explicit cargoes for degradation within lysosomes when cells are exposed to various stresses like DNA damage (52, 53). However, during the development of NAFLD pathology, selective autophagy primarily plays a role in degrading LDs through mitophagy and ER-phagy processes (57).

The pathogenesis of NAFLD is intricate and primarily associated with the interplay of steatosis-induced inflammation, lipotoxicity, oxidative stress, and the ER (8). During disease progression, excessive accumulation of fat in hepatocytes, increased levels of FFAs and TG stimulate hepatocyte steatosis, induce mitochondrial dysfunction, and ERS. This promotes excessive production of ROS, impairs autophagy, promotes the capillarization of LSECs, and stimulates the activation of inflammasomes in KCs. Simultaneously, it also enhances the secretion of fibrotic factors that stimulate the activation of adjacent HSCs and facilitate the progression of NAFLD to liver fibrosis (126). However, autophagy has a dual role in HSCs, promoting the growth of quiescent HSCs, while inhibiting the growth of activated HSCs. This prevents fibrosis changes in NAFLD and its involvement in all stages of NAFLD progression (115). In recent years, there have been reports discussing drugs that target autophagy for the treatment of NAFLD, including herbal extracts, emerging GLP-1 activators, and SGLT2 inhibitors (91). These drugs have demonstrated their ability to induce or restore the process of autophagy, ameliorate hepatocyte steatosis, reduce inflammation caused by KCs, inhibit HSC activation, decrease the occurrence of liver fibrosis, and delay the progression of NAFLD (115).

Three types of autophagy have been identified, namely microautophagy, macroautophagy, and CMA. However, numerous unresolved questions remain regarding the intricate interplay among these processes and the specific triggering mechanisms involved in autophagy. Unresolved inquiries persist concerning identification of novel receptors involved in selective autophagy for autophagy-mediated degradation. Despite extensive knowledge of liver autophagy, the specific mechanism of action of drugs targeting autophagy in the pathogenesis of NAFLD remains to be determined. Certain drugs have not transitioned from animal experiments to clinical practice due to a lack of theoretical support from human clinical research. Similarly, the current research stage in NAFLD pathology lacks sufficient investigation into drug targeting of autophagy through signaling pathways, specifically in hepatic sinusoidal endothelial cells and cholangiocytes. In the future, by regulating the role of autophagy through signaling pathways, we will be able to identify better therapeutic targets to treat NAFLD more effectively.

## 6 Conclusions

The activation of autophagy can inhibit hepatocyte steatosis, suppress the production of inflammatory factors in KCs, prevent the activation of HSCs, and maintain LSECs. In contrast, impaired autophagy is involved in different stages of NAFLD. However, despite ongoing clinical studies and the use of some drugs to improve autophagy in NAFLD patients, the incidence of this disease continues to rise. The maintenance of autophagy in innate liver cells is an urgent problem that needs to be addressed in the medical field. This study aims to provide novel perspectives and insights for the clinical management of NAFLD by summarizing the physiological process of autophagy, elucidating the association between autophagy and NAFLD, and exploring the role and relevant pathways of intracellular therapeutic agents.

## Author contributions

QS: Writing – original draft, Writing – review & editing, Conceptualization, Project administration, Visualization. MY: Writing – review & editing, Project administration, Visualization. SW: Investigation, Writing – review & editing. XC: Writing – original draft, Investigation, Project administration. SC: Writing – original draft, Project administration. RZ: Investigation, Writing – original draft. ZX: Writing – review & editing, Visualization. YL: Supervision, Writing – review & editing.
