# Supplementary material for: The pivotal role of dysregulated autophagy in the progression of non-alcoholic fatty liver disease
Source: Front Endocrinol (Lausanne). 2024 Aug 8;15:1374644. doi: 10.3389/fendo.2024.1374644 (PMC11338765; doi:10.3389/fendo.2024.1374644)
Supplement: Supplementary file 1 [file DataSheet_1.docx]

Supplementary Material

# Supplementary Figures and Tables

## Supplementary Figures


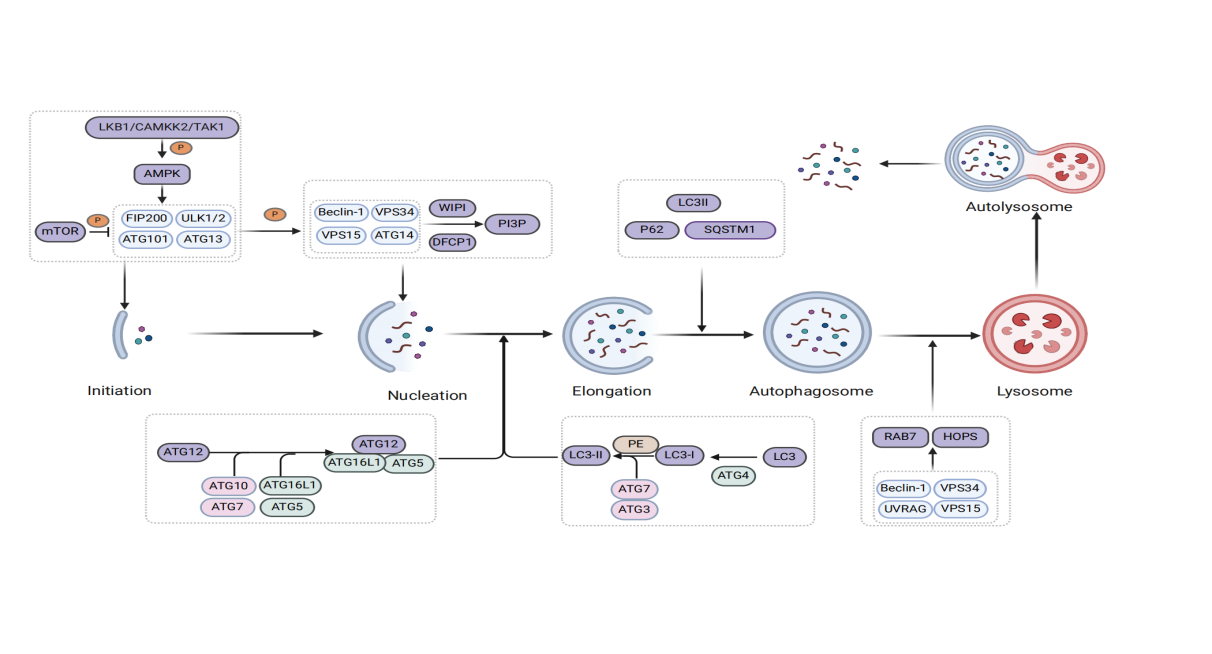


## Supplementary Figure 1 The biological process of autophagy (Created with BioRender.com).


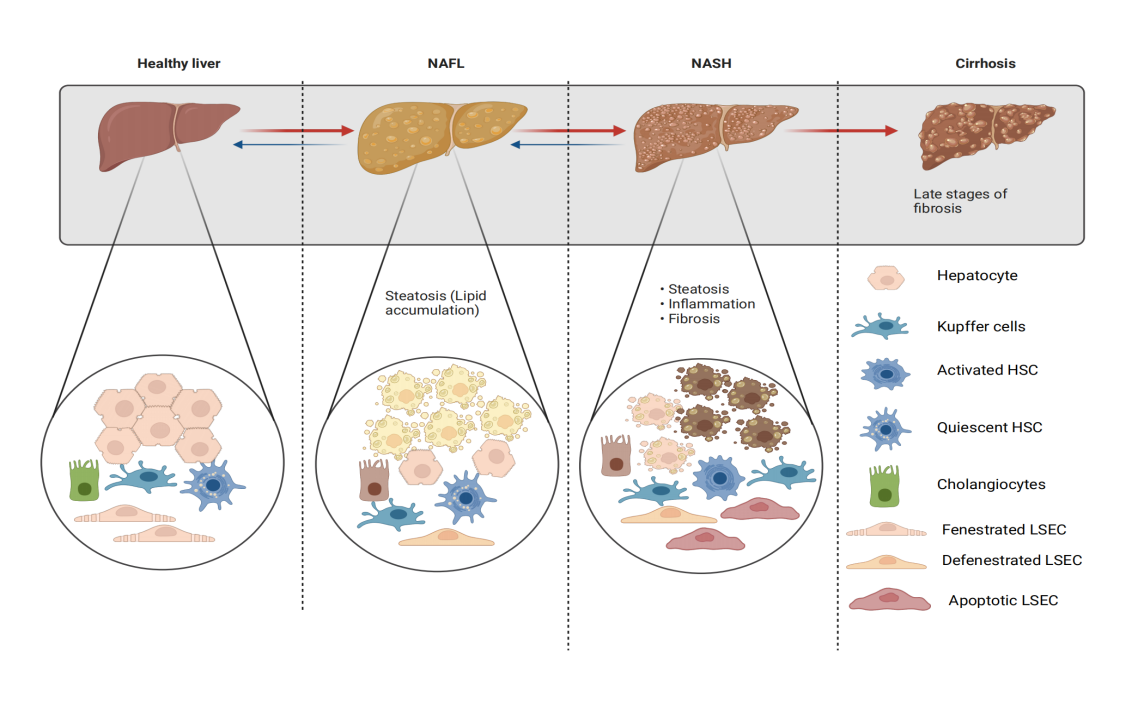


## Supplementary Figure 2 Changes of liver parenchyma and non-parenchymal cells in NAFLD (Created with BioRender.com). The transition between fenestrated and defenestrated LSECs: under physiological conditions, LSECs are perforated by fenestrations and lack a basement membrane; however, under pathological conditions, LSECs lose their fenestrations and form a continuous basement membrane. This phenomenon is called "capillarization". Capillarization, i.e. loss of LSECs fenestrae, and LSECs dysfunction, i.e. the loss of the ability of LSECs to generate vasodilator agents in response to increased shear stress, are two events occurring early in NAFLD. The transition from quiescent to active HSCs: a distinct feature of quiescent hepatic stellate cells is the storage of retinoids (vitamin A and its metabolites) within their cytoplasmic lipid droplets. However, activated hepatic stellate cells exhibit a contractile, proliferative, and fibrogenic phenotype, which can be further distinguished from quiescent hepatic stellate cells by the loss of their retinol-containing lipid droplets.


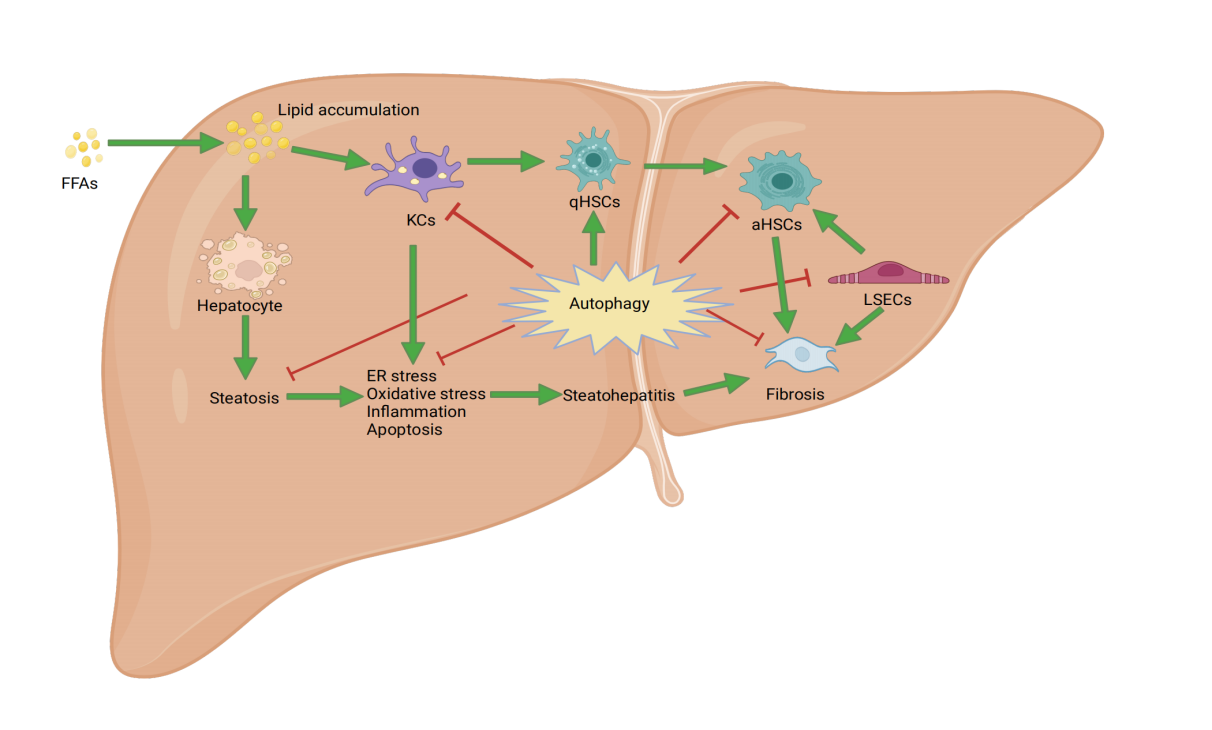


**Supplementary Figure 3** Mechanism of autophagy in nonalcoholic fatty liver disease. FFAs: free fatty acids; ER: endoplasmic reticulum stress; aHSCs: activated hepatic stellate cells;qHSCs: Quiescent hepatic stellate cells; KCs: Kupffer cells, LSECs: liver sinusoidal endothelial cells(Created with BioRender.com).


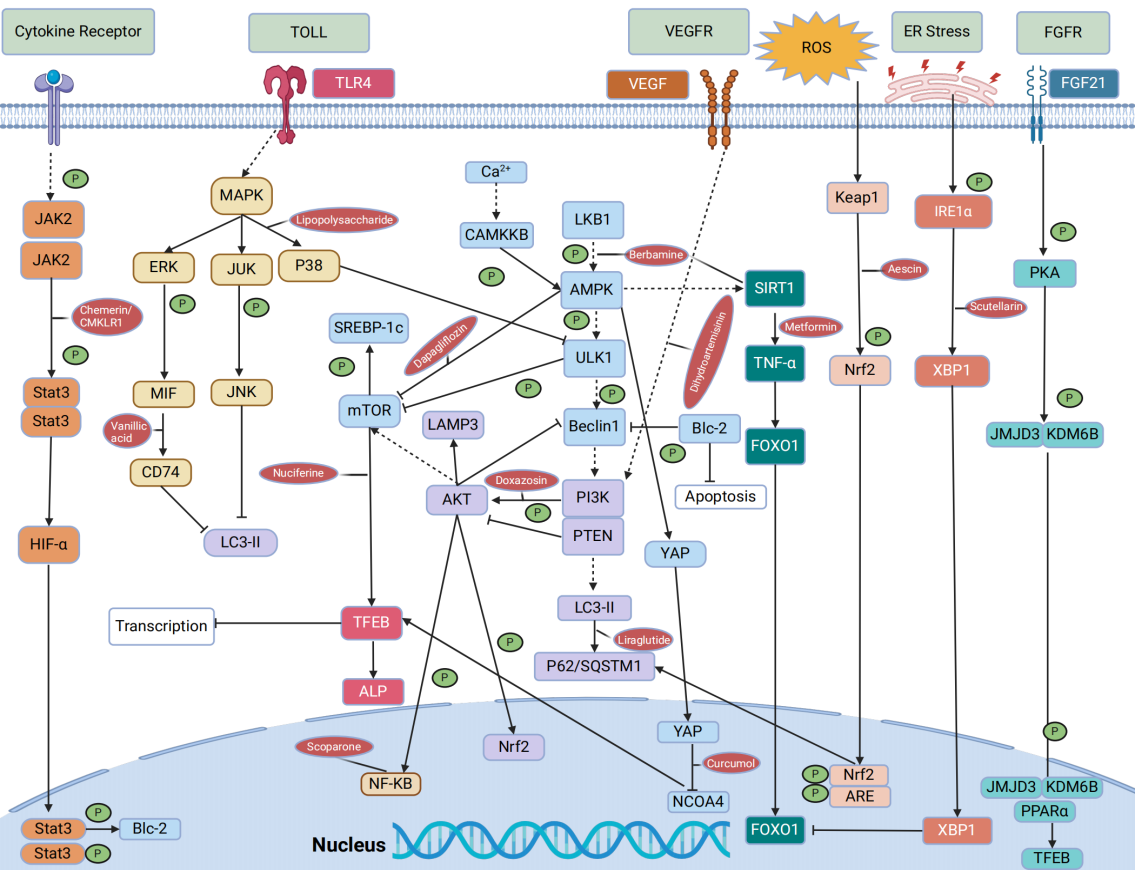


**Supplementary Figure 4.** Signaling pathways that regulate autophagy or are regulated by autophagy in NAFLD. (Created with BioRender.com).

## Supplementary Tables

**Table 1 The role of autophagosome formation in NAFLD(drugs with clinical trials)**

| Treatment | Signaling pathway | Modeling method (animal/cell) | Conclusion | References |
| --- | --- | --- | --- | --- |
| Berbamine(BBM) | SIRT1/LKB1/AMPK signaling pathway | Wistar rats | BBM mitigates liver lipid metabolism disorders by modulating the SIRT1/LKB1/AMPK pathway, regulating the expression of autophagy markers LC3a/b, Beclin 1, and p62, and inducing autophagy to decelerate the progression of NAFLD. | 144 |
| Dapagliflozin | AMPK-mTOR  signaling pathway | ZDF rats and ZL rats; PA-induced LO2 and HepG2 cells | Dapagliflozin affects hepatic steatosis in NAFLD by downregulating lipogenic enzymes and activating the AMPK-mTOR pathway, while promoting fatty acid oxidation and autophagy to mitigate disease progression. | 145 |
| Doxazosin | PI3K/Akt/mTOR signaling pathway | CCl4 induced C57BL/6J mice；LX-2 cells | Doxazosin inhibits autophagy by activating the PI3K/Akt/mTOR signaling pathway, attenuating liver fibrosis. | 178 |
| Empagliflozin | AMPK/mTOR signaling pathway | HFD-fed ApoE(-/-) mice | Empagliflozin activates the AMPK/mTOR pathway, upregulates LC3B expression, induces autophagy, and ameliorates NAFLD | 142 |
| Empagliflozin | AMPK/mTOR signaling pathway | HFD induced C57BL/6J mice | Empagliflozin significantly enhances the autophagy of liver macrophages through the AMPK/mTOR signaling pathway, inhibits the expression level of IL-17/IL-23 axis-related molecules, reduces inflammatory response, and improves NAFLD-related liver injury. | 169 |
| Imatinib | STAT3/IL-6 signaling pathway | LX-2 cell;CCl4 induced SD rats | Imatinib suppresses the activation of HSCs through the specific targeting of the STAT3/IL-6 pathway via miR-124. | 180 |
| Liraglutide（LRG） | AMPK/mTOR/Beclin1 signaling pathway | HFD induced C57BL/6 mice；FFA induced L-O2 cells | LRG induces autophagy through the AMPK/mTOR/Beclin1 pathway, regulating the expression of autophagy proteins SQSTM1/P62 and LC3B, thereby improving hepatic lipid accumulation in NAFLD. | 149 |
| Metformin | PRKA/SIRT1/FOXO signaling pathway | ob/ob mice | Metformin targets PRKA to activate the SIRT1/FOXO signaling pathway, thereby inducing autophagy and slowing down the progression of NAFLD. | 143 |
| Metformin | AMPK-Sirt1 signaling pathway | Ttp mice;KCs;  PHH | Metformin activates TTP through the AMPK-Sirt1 pathway, inhibits the production of KCs to suppress TNF-α, downregulates Rheb expression, inhibits mTORC1 expression, enhances TFEB nuclear translocation, and promotes autophagy. | 165 |
| Scoparone | ROS/P38/Nrf2 axis and PI3K/AKT/mTOR signaling pathway | MCD induced C57BL/6；LPS induced RAW264.7 and PA-induced AML12 cells | Scoparone can ameliorate liver inflammation and enhance autophagy in NASH mice by inhibiting the ROS/P38/Nrf2 axis and PI3K/AKT/mTOR pathway, thereby promoting autophagic flux while suppressing inflammation. | 162 |
| Scoparone | TLR4/NF-κB signaling pathway | MCD induced C57BL/6 mice；RAW264.7 cells | Scoparone mitigates inflammation, apoptosis, and fibrosis in NASH by suppressing mice's TLR4/NF-κB signaling pathway. | 163 |
| Ursodeoxycholic acid (UDCA) | Beclin-1-Bcl-2 complex-mediated signaling pathway | HFD induced SD rats | UDCA modulates the interaction between the Bcl-2/Beclin-1 complex and the Bcl-2/Bax complex through activation of the AMPK pathway, thereby inducing autophagy and impeding the progression of NAFLD. | 151 |
| Verapamil | mTOR signaling pathway | HFD indeced C57BL/6 mice | Verapamil induces autophagy through an mTOR-independent signaling pathway, improves hepatocyte function, and promotes hepatocyte regeneration. | 161 |

NAFLD, non-alcoholic fatty liver disease; SIRT1, sirtuin1; LKB1, liver kinase B1; AMPK, AMP-activated protein kinase; mTOR, mammalian target of rapamycin; ZDF rats, Zucker Diabetic Fatty rats; ZL rats, Zucker lean rats; PA, palmitic acid; LO2, human normal liver cell; HepG2, human hepatocellular carcinomas; CCl4, carbon tetrachloride; LX-2 cells, human hepatic stellate cells; PI3K, phosphatidylinositol three kinase; AKT, protein kinase B; HFD, high fiber diet; CCl4, carbon tetrachloride; SD, Sprague-Dawley rats; FFA, free fatty acid; IL-17, interleukin 17; IL-23, interleukin,23; STAT3, signal transducer and activator of transcription 3; IL-6, interleukin 6; PRKA, AMP-activated protein kinase; FOXO, forkhead box O; ob/ob mice, obese mice; PHH, primary hepatocyte model; TTP, tristetraprolin;TNF-α, tumor necrosis factor-α; KCs,kupffter cells; TFEB, transcription factor EB; Nrf2, Nuclear factor erythroid2-related factor 2; MCD, methionne-choline deficient diet; LPS, lipopolysaccharide; RAW 264.7 cells , mouse mononuclear macrophages cells; AML12 cells, alpha mouse liver 12 cells; TLR4, toll-like receptor 4; NF-κB, nuclear factor kappa-B;NASH, nonalcoholic steatohepatitis.

**Table 2 The role of autophagosome formation in NAFLD (drugs without clinical trials).**

| Treatment | Signaling pathway | Modeling method (animal/cell) | Conclusion | References |
| --- | --- | --- | --- | --- |
| Acetylshikonin（AS） | AMPK/mTOR signaling pathway | MCD induced C57BL/6 mice | AS enhances hepatocyte autophagy via the AMPK/mTOR pathway to ameliorate NAFLD. | 141 |
| Aescin (Aes) | Keap1-Nrf2 signaling pathway | Nrf2/C57BL/6 mice；Atg5/C57BL/6 mice | Aescin effectively alleviated NAFLD by regulating the Keap1-Nrf2 pathway and activating antioxidant mechanisms and autophagy | 155 |
| Alisol A 24-acetate | AMPK/mTOR/ULK1 signaling pathway | LX-2 cells;MCD induced C57BL/6 mice | Alisol A 24-acetate exerts its therapeutic effects on NASH by activating autophagy via the AMPK/mTOR/ULK1 pathway, suppressing the expression of pro-inflammatory cytokines and reactive ROS, and alleviating oxidative stress. | 176 |
| Curcumol | YAP/NCOA4 axis | C57BL/6J mice;LO2 cells | Curcumol inhibits hepatocyte senescence by regulating NAFLD iron autophagy through YAP/NCOA4. | 157 |
| Curcumin derivative | AMPK/TGF-β signaling pathway | TGF-β induces LX-2 cells and hepatocytes (alpha mouse liver 12 [AML12]) | Curcumin derivatives combined with TGF-β receptor I inhibitors can attenuate liver fibrosis and impede the progression of NAFLD. | 174 |
| Curcumin | PI3K/Akt/mTOR signaling pathway | HSCs line LX-2 cells | The activation of the PI3K/Akt/mTOR signaling pathway by curcumin can effectively inhibit autophagy, suppress the activity, and induce apoptosis in LX-2 cells derived from the HSCs line, thereby attenuating the progression of liver fibrosis. | 175 |
| Dihydroartemisinin | VEGF/PI3K/AKT/mTOR/ULK1 signaling pathway | SD rats；HSC‑LX2 cells | The regulation of autophagy by dihydroartemisinin involves the modulation of the VEGF pathway and mediation of the PI3K/AKT/mTOR/ULK1 pathway, leading to inhibition of HSCs activation and delayed progression of NAFLD. | 177 |
| Ginsenoside Rg1 | PTEN-AKT signaling pathway | FFA induced HepG2；MCD induced C57BL/6 mice | Ginsenoside Rg1 exerts its effects on autophagy and pyroptosis by modulating the miR-375-3p/ATG2B/PTEN-AKT pathway, thereby mitigating the pathogenesis and progression of NAFLD. | 147 |
| Glycyrrhetinic acid | STAT3-HIF-1α signaling pathway | C57BL/6 mice;KCs cells | Glycyrrhetinic acid modulates the STAT3-HIF-1α pathway in macrophages, enhancing autophagy flux impairment. This mitigates the excessive generation of inflammatory cytokines and hepatocyte apoptosis, thereby alleviating the progression of NAFLD. | 168 |
| Icaritin | AMPK signaling pathway | sodium oleate-induced L02 and Huh-7 cells | Icaritin attenuates lipid accumulation by increasing energy expenditure and autophagy regulated by phosphorylating AMPK. | 159 |
| Lipopolysaccharide(LPS) | MAPK p38/Ulk1 signaling pathway | LPS-induced HSC-T6 cells | The expression of IL-1β induced by autophagy in HSCs is inhibited by regulating the MAPK p38/Ulk1 pathway in LPS-induced HSC-T6 cells. | 182 |
| Magnolol(MG) | Nrf2-ARE and mTOR signaling pathway | PA-induced HepG2; Tyloxapo-induced Wistar rats | MG inhibits mTOR and activates the Nrf2-ARE pathway, enhancing autophagic flux and ameliorating hepatocyte steatosis. | 156 |
| Pueraria flavonoids | PI3K/Akt/mTOR signaling pathway | C57BL/6J mice were induced by 40% fat; PA-induced HepG2 cells | Pueraria radix flavonoids induce autophagy by inhibiting the PI3K/Akt/mTOR signaling pathway, thereby reducing intracellular lipid accumulation and inflammation levels, ultimately ameliorating NAFLD. | 146 |
| Palmitic acid | Hh signaling | LX2、HSCs and rat BSC-C10 cells | The activation of HSCs is induced by palmitic acid through the inflammasome and Hh signaling pathways. | 181 |
| Resveratrol | SIRT1 and JNK signaling pathway | Immortalized mouse HSC line JS1 cells | Resveratrol modulates autophagy and apoptosis via the SIRT1 and JNK signaling pathways, suppresses HSCs activation, and attenuates liver fibrosis in NAFLD. | 171 |
| Schisandrin B(Sch B) | AMPK/mTOR signaling pathway | FFA induced HepG2；HFD induced C57BL/6J mice | Sch B activates autophagy through the AMPK/mTOR pathway, inhibits steatosis, and promotes fatty acid oxidation, thereby alleviating the progression of NAFLD. | 140 |
| Soluble epoxide hydrolase(sEH)/cyclooxygenase-2(COX-2) Dual Inhibitor | Sirt1/PI3K/AKT/mTOR signaling pathway | HFD induced C57BL/6J mouse;PA-induced AML12 hepatocytes | The COX-2/EH complex can suppress the PI3K/AKT/mTOR signaling pathway via Sirt1, enhancing autophagy, decelerating hepatocyte senescence, and ameliorating NAFLD. | 148 |
| Scutellarin (Scu) | IRE1α/XBP1/FoxO1 signaling pathway | PA-induced HepG2 cells;HFD induced C57/BL6 mice | Scu down-regulates SREBP-1c expression through the IRE1α/XBP1/FoxO1 pathway, inhibits endoplasmic reticulum stress, and enhances autophagy to improve liver lipid accumulation. | 160 |
| Salvianolic acid B | MAPK/p38/JUK signaling pathway | JS1 and LX2 cells | The MAPK pathway is down-regulated by salvianolic acid B to inhibit TGF-β1-induced autophagy and activation of HSCs. | 172 |
| Vanillic acid | MIF/CD74 signaling pathway | ccl4 induced SD  rats;HSCs-T6cells | The progression of NAFLD to liver fibrosis can be alleviated by vanillic acid, which inhibits the autophagy of HSCs through the MIF/CD74 signaling pathway. | 173 |
| 1,3-dichloro-2-propanol (1,3-DCP) | AKT/mTOR/FOXO1 signaling pathway | C57BL/6 mice; HepG2 cells | 1, 3-DCP activates the phosphorylation of AKT and mTOR, reduces the expression of FOXO1, and inhibits autophagy-mediated lipid accumulation | 158 |

Keap1, kelch-like ECH-associated protein-1; ULK1, unc-51-like kinase 1; YAP, Yes-associated protein; NCOA4, nuclear receptor coactivator 4; TGF-β, Transforming growth factor beta; HSCs, hepatic stellate cells; VEGF, vascular endothelial-derived growth factor; PTEN, phosphatase and tensin homolog; HIF-1α, hypoxia-inducible factor-1α; MAPK, mitogen-activated protein kinase; ARE, anti-oxidative response element; Hh,Hedgehog; JS1, the mouse immortalized stellate cell lines; JNK, Jun N-terminal kinase; IRE1α/XBP1, inositol-requiring enzyme 1α(IRE1α)/ X-box-binding protein 1 (XBP1); FOXO1, Forkhead box O1; MIF, migration inhibitory factor;

**Table 3 The role of autophagosome formation in NAFLD (low or high expression of a particular target).**

| Treatment | Signaling pathway | Modeling method (animal/cell) | Conclusion | References |
| --- | --- | --- | --- | --- |
| Acetaminophen (APAP) | AMPK/mTOR/SREBP-1c signaling pathway | C57BL/6J mice;L02 cells | The overdose of APAP reduces LC3-II and Beclin1 levels through the AMPK/mTOR/SREBP-1c pathway, inhibiting autophagy and exacerbating the progression of NAFLD. | 152 |
| Angiotensin-converting enzyme 2（ACE2）overexpression | AMPK/mTOR signaling pathway | rAAV2/8-ACE2 induced C57 mice； | Overexpression of the ACE2 protein activates the AMPK/mTOR pathway, modulates HSCs autophagy, suppresses HSCs activation, and decelerates the progression of liver fibrosis in individuals with NAFLD. | 179 |
| Chemerin/CMKLR1 | JAK2-STAT3  signaling pathway | HFD-induced C57BL/6 mice | The regulation of the JAK2-STAT3 pathway by Chemerin/CMKLR1 can augment autophagy and ameliorate hepatic oxidative stress, thereby enhancing NASH. | 153 |
| Jumonji-D3 （JMJD3/KDM6B） | PPARα signaling pathway | HFD induced JMJD3-floxed mice;Hepa1c1c7 cells | FGF21 signaling triggers the PPARα pathway through JMJD3/KDM6B histone demethylase, promoting hepatic autophagy and hepatocyte lipid degradation in NAFLD. | 154 |
| Lp-PLA2 low expression | JAK2/STAT3 signaling pathway | HFD induced C57BL/6J mice；KCs | The down-regulation of Lp-PLA2 expression inhibits the JAK2/STAT3 signaling pathway, reduces lipid accumulation, promotes autophagy, suppresses the production of inflammatory factors, and delays the progression of NAFLD. | 164 |
| Maresin 1 | AMPK-SERCA2b signaling pathway | HFD-induced C57 mice | Maresin 1 mitigates NAFLD by attenuating endoplasmic reticulum stress via the AMPK-SERCA2b pathway. | 170 |
| TIM-4 | Akt4/Mitophagy signaling pathway | C57 mice;KCs | The inhibition of CCL14-induced liver fibrosis by TIM-4 is achieved through interference with KCs via the Akt4/mitophagic signaling pathway, and it also delays the progression of NAFLD. | 166 |
| Tim-4 | LKB3/AMPKα signaling pathway | C57BL/6 mice; KCs and HSCs | The inhibition of the NLRP1 inflammasome by Tim-4 in liver macrophages through the LKB3/AMPKα pathway serves as an intervention strategy for NAFLD progression. | 167 |

rAAV2/8-ACE2,recombinant adeno-associated virus ACE2 vector; JAK2-STAT3, the Janus kinase (JAK)2/signal transductors and the transcription (STAT)3; Chemerin/CMKLR1, chemerin chemokine-like receptor 1 Gene; PPARα, peroxisome proliferator-activated receptor-α; FGF21, fibroblast growth factor; JMJD3,Jumonji domain-containing protein D3; Hepa1c1c7 cells, mouse hepatocarcinoma cells.

**Table 4 The role of autolysosome formation in NAFLD**

| Treatment | Signaling pathway | Modeling method (animal/cell) | Conclusion | References |
| --- | --- | --- | --- | --- |
| Ajugol | mTOR/TFEB signaling pathway | PA-induced hepatocytes;HFD induced C57BL/6J mice; | Ajugol inhibits mammalian targets of mTOR and induces nuclear translocation of TFEB, thereby promoting the TFEB-mediated autophagy-lysosomal pathway and lipo autophagy to ameliorate NAFLD. | 191 |
| Baicalein | mTOR signaling pathway | PA-induced HepG2;HFD induced C57BL/6J mice; | Baicalein can inhibit the mTOR signaling pathway and improve lysosomal membrane permeability in treating NAFLD. | 190 |
| Fenofibrate | CaMKKβ-AMPK-ULK1 signaling pathway | HFD induced mice；HepG2 | The CaMKKβ-AMPK-ULK1 signaling pathway is regulated by fenofibrate, leading to the activation of lysosomal Ca^2+^ levels, induction of TFEB activation, promotion of fat phagocytosis, and ultimately improvement in fat accumulation in NAFLD. | 184 |
| Jiang Zhi Granule | PI3K-AKT-mTOR signaling pathway | PA induced HepG2;HFD induced C57BL/6J mice; | The Jiangzhi granule induces either autophagosome formation or the co-localization of autophagosomes and lysosomes through the PI3K-AKT-mTOR signaling pathway, facilitating the degradation of autophagosomes to prevent hepatocyte injury effectively. | 189 |
| Liraglutide | TFEB-mediated  signaling pathway | HFD induced  C57BL/6J mic  e; HepG2 | Liraglutide ameliorates hepatic steatosis by inducing activation of the TFEB-mediated ALP through modulation of LC3-II and SQSTM1/P62 autophagy substrates expression levels. | 183 |
| Lysosome-associated membrane protein 3（LAMP3） | PI3K/Akt signaling pathway | FFA induced HCC cells;HFD induced C57BL/6J mice;ob/ob mice | The overexpression of LAMP3 induces activation of the PI3K/Akt pathway, reducing TG content and promoting autolysosome fusion. | 188 |
| Nuciferine | mTORC1-TFEB-ALP signaling pathway | HFD induced C57BL/6J mice; HepG2 | Nuciferine modulates hepatic steatosis and insulin resistance via the mTORC1-TFEB-ALP axis to attenuate the progression of NAFLD. | 185 |
| Phillygenin | Ca^2+^calcineurin-TFEB axis | PA-induced AML12 cells and primary hepatocytes;HFD induced C57BL/6J mice; | Phillygenin exerts a regulatory effect on the Ca^2+^ calcineurin-TFEB axis, enhancing lysosomal biogenesis and autophagy flux in hepatocytes and reducing lipid deposition. | 187 |
| Quercetin | IRE1a/XBP1s signaling pathway | HFD-induced SD rats; FFA-induced HepG2 cells | The activation of IRE1a/XBP1s by quercetin effectively enhances the colocalization of lysosomes with lipid droplets, reduces p62 accumulation, and promotes liver fat autophagy in NAFLD. | 186 |

ALP, autophagy-lysosomal pathway; CaMKKβ, calcium/calmodulin-dependent protein kinase kinase β protein.
